# Exposure to flame retardants in European children — Results from the HBM4EU aligned studies

**DOI:** 10.1016/j.ijheh.2022.114070

**Published:** 2023-01

**Authors:** Veronica van der Schyff, Jiři Kalina, Eva Govarts, Liese Gilles, Greet Schoeters, Argelia Castaño, Marta Esteban-López, Jiři Kohoutek, Petr Kukučka, Adrian Covaci, Gudrun Koppen, Lenka Andrýsková, Pavel Piler, Jana Klánová, Tina Kold Jensen, Loic Rambaud, Margaux Riou, Marja Lamoree, Marike Kolossa-Gehring, Nina Vogel, Till Weber, Thomas Göen, Catherine Gabriel, Dimosthenis A. Sarigiannis, Amrit Kaur Sakhi, Line Småstuen Haug, Lubica Palkovicova Murinova, Lucia Fabelova, Janja Snoj Tratnik, Darja Mazej, Lisa Melymuk

**Affiliations:** a RECETOX, Faculty of Science, Masaryk University, Kotlarska 2, Brno, Czech Republic; b VITO Health, Flemish Institute for Technological Research (VITO), Mol, 2400, Belgium; c Department of Biomedical Sciences, University of Antwerp, 2020, Antwerp, Belgium; d National Centre for Environmental Health, Instituto de Salud Carlos III, Majadahonda, Madrid, Spain; e Toxicological Center, University of Antwerp, 2610 Wilrijk, Belgium; f Department of Environmental Medicine, Institute of Public Health, University of Southern Denmark, Odense, 5000, Denmark; g Santé Publique France, French Public Health Agency (ANSP), Saint-Maurice, 94415, France; h Vrije Universiteit, Amsterdam Institute for Life and Environment, Section Chemistry for Environment & Health, De Boelelaan 1108, 1081 HZ, Amsterdam, Netherlands; i German Environment Agency (UBA), 06844 Dessau-Roßlau, Germany; j IPASUM - Institute and Outpatient Clinic of Occupational, Social and Environmental Medicine, Henkestrasse 9-11, 91054, Erlangen, Germany; k Environmental Engineering Laboratory, Department of Chemical Engineering, Aristotle University of Thessaloniki, 54124, Thessaloniki, Greece; l HERACLES Research Center on the Exposome and Health, Center for Interdisciplinary Research and Innovation, Balkan Center, Bldg. B, 10th km Thessaloniki-Thermi Road, 57001, Greece; m Environmental Health Engineering, Institute of Advanced Study, Palazzo del Broletto, Piazza Della Vittoria 15, 27100, Pavia, Italy; n Department of Environmental Health, Norwegian Institute of Public Health, Oslo, Norway; o Faculty of Public Health, Slovak Medical University, Bratislava, 833 03, Slovakia; p Department of Environmental Sciences, Jožef Stefan Institute, Ljubljana, 1000, Slovenia

**Keywords:** Polybrominated diphenyl ethers, Organophosphate flame retardants, Children, Europe, HBM4EU, Human biomonitoring

## Abstract

Many legacy and emerging flame retardants (FRs) have adverse human and environmental health effects. This study reports legacy and emerging FRs in children from nine European countries from the HBM4EU aligned studies. Studies from Belgium, Czech Republic, Germany, Denmark, France, Greece, Slovenia, Slovakia, and Norway conducted between 2014 and 2021 provided data on FRs in blood and urine from 2136 children. All samples were collected and analyzed in alignment with the HBM4EU protocols. Ten halogenated FRs were quantified in blood, and four organophosphate flame retardants (OPFR) metabolites quantified in urine. Hexabromocyclododecane (HBCDD) and decabromodiphenyl ethane (DBDPE) were infrequently detected (<16% of samples). BDE-47 was quantified in blood from Greece, France, and Norway, with France (0.36 ng/g lipid) having the highest concentrations. BDE-153 and -209 were detected in <40% of samples. Dechlorane Plus (DP) was quantified in blood from four countries, with notably high median concentrations of 16 ng/g lipid in Slovenian children. OPFR metabolites had a higher detection frequency than other halogenated FRs. Diphenyl phosphate (DPHP) was quantified in 99% of samples across 8 countries at levels ∼5 times higher than other OPFR metabolites (highest median in Slovenia of 2.43 ng/g lipid). FR concentrations were associated with lifestyle factors such as cleaning frequency, employment status of the father of the household, and renovation status of the house, among others. The concentrations of BDE-47 in children from this study were similar to or lower than FRs found in adult matrices in previous studies, suggesting lower recent exposure and effectiveness of PBDE restrictions.

## Highlights

• Flame retardants quantified in blood and urine from children from nine countries.
• DPHP and BDCIPP had the highest detection frequencies.
• Multiple lifestyle factors were associated with flame retardant concentrations.
• Evidence of widespread exposure of European children to OPFRs.

## 1 Introduction

Human biomonitoring (HBM) is the analysis of substances and/or their respective metabolites in human matrices. HBM is a crucial tool to evaluate and monitor internal chemical exposure in specific population samples, identify chemicals of concern, study determinants of exposure, or evaluate the efficacy of regulations in place to mitigate chemical exposure (Angerer et al., 2007, 2011).

Children often have elevated exposure to many chemicals (Gibson et al., 2019; Koppen et al., 2019), including industrial compounds such as flame retardants (FRs), due to an increased inhalation rate, different breathing zone, increased hand-to-mouth activity, and faster metabolism (van den Eede et al., 2015). Several FRs are known endocrine disruptors that disrupt thyroid function in children, resulting in adverse effects on the individual's long-term mental health, cognitive ability, metabolism, and reproduction (Preston et al., 2017). Since children are the most vulnerable age group to chemical exposure, they should be a priority for human and environmental health policy (Au, 2002).

The European Human Biomonitoring Initiative (HBM4EU) is a large-scale HBM project co-funded under the European Commission's Research and Innovation Program Horizon 2020 that includes 30 European countries and the 10.13039/501100000806European Environment Agency. One of its major aims is to enhance the body of evidence of European citizens' internal exposure to chemicals (David et al., 2020; Lange et al., 2021). Flame retardants were identified by European Union (EU) institutions and HBM4EU partner countries as priority substances to be studied as knowledge gaps with an impact on regulation still exist (Louro et al., 2019). Several detrimental ecological and human health issues have been associated with elevated FR concentrations, including environmental persistence, long-range transport, bioaccumulation, and endocrine and neurologically disruptive effects on organisms, including humans (Aznar-Alemany et al., 2019; Bajard et al., 2019, 2021; Stieger et al., 2014; Sverko et al., 2011)

Flame retardants have been widely used since the 1970s in textiles, furnishing, plastic, and electronic equipment (Horrocks, 2011; McGrath et al., 2018; Morel et al., 2022). Brominated flame retardants (BFRs), including polybrominated diphenyl ethers (PBDEs) and hexabromocyclododecanes (HBCDDs), were the primary FRs for more than thirty years (Pantelaki and Voutsa, 2019). PBDEs and HBCDDs have been strictly regulated by several international bodies, including the Registration, Evaluation, Authorization, and Restriction of Chemicals (REACH), the United States Environmental Protection Agency (USEPA), and the Stockholm Convention (Guo et al., 2016; Kemmlein et al., 2009; Sharkey et al., 2020) since 2004. These compounds are often referred to as legacy FRs given their limited new use. Since the restrictions on the use and production of legacy FRs came into force, the production of alternative FRs, such as organophosphate flame retardants (OPFRs) and novel halogenated flame retardants (NFRs), has increased (Covaci et al., 2011; He et al., 2021). Some commonly used halogenated NFRs are decabromodiphenyl ethane (DBDPE), bis(2-ethylhexyl) tetrabromophthalate (BEH-TEBP), tetrabromobisphenol A (TBBPA) and Dechlorane Plus (DP). Collectively, NFRs and OPFRs are referred to as emerging FRs.

Because OPFRs are less persistent than legacy FRs and have shorter elimination half-lives, it was assumed that these compounds are less harmful to human and environmental health (Blum et al., 2019). However, this assumption has recently been questioned, as several OPFRs are associated with endocrine disruptive effects at the same levels as PBDEs (Behl et al., 2016).

Since the twin projects COPHES (Consortium to Perform Human Biomonitoring on a European Scale) (Joas et al., 2012) and the feasibility study DEMOCOPHES (DEMOnstration of a study to COordinate and Perform Human Biomonitoring on a European Scale) from 2009 to 2012 (Den Hond et al., 2015), no large-scale multi-country HBM project has been conducted to determine chemical compound concentrations in the biological matrices of children or quantified emerging FRs in children. Our study aims to quantify legacy and emerging FRs in children from nine European countries through information obtained by the HBM4EU aligned studies. This activity will enable concentrations of different countries to be comparable to each other. To understand the potential sources of flame retardant exposure, several lifestyle factors were investigated based on ancillary data gathered by questionnaires.

## 2 Materials and methods

### 2.1 Study alignment and participation

One of the aims of the HBM4EU aligned studies is to harmonize chemical and data analyses of human biological samples throughout Europe (Ganzleben et al., 2017; Gilles et al., 2021). The project builds on existing capacities within the EU by aligning already existing studies targeting different populations or regions (Gilles et al., 2021). Eight countries and six HBM4EU qualified laboratories were involved in the study of FRs in children (Table 1). The CELSPAC cohort from the Czech Republic was not part of the HBM4EU study. However, the collection, analyses, and QA/QC protocols were aligned with the HBM4EU study as other CELSPAC age groups were used for HBM4EU aligned studies, thus the data collected from the CELSPAC study can be compared with the cohorts participating in the HBM4EU study.

**Table 1:** Descriptive statistics of the participants of the study and the compounds analyzed in each country.

COUNTRY (COUNTRY CODE)^a^	STUDY	TOTAL PARTICIPANTS	FEMALE	MALE	AGE RANGE (YEARS)	SAMPLING YEARS	COMPOUNDS ANALYZED	STUDY REFERENCE, WHERE AVAILABLE
BELGIUM (BE)	3xG	133	67	66	7–8	2019–2020	BDCIPP, DPHP, BCIPP	Govarts et al. (2020)
CZECH REPUBLIC (CZ)	CELSPAC	195	106	89	11–12	2019	BCEP, BCIPP. BDCIPP, DPHP	Not available
GERMANY (DE)	GerES V	300	150	150	6–12	2015–2016	BCEP, BCIPP, BDCIPP, DPHP	European Commission (2021a)
DENMARK (DK)	OCC	291	130	161	7	2018–2019	BDCIPP, DPHP, BCIPP	Kyhl et al. (2015)
GREECE (EL)	CROME	55	31	24	6–11	2020–2021	PBDE-47, -153, −209, DP, HBCDD, DBDPE	(EnvE Lab, 2022; Gilles et al., 2022)
FRANCE (FR)	ESTEBAN	413	191	222	7–13	2014–2016	PBDE-47, -153, −209, DP, HBCDD, DBDPE, BEH-TEBP, TBBPA, BCIPP, BDCIPP, DPHP	European Commission (2021b)
NORWAY (NO)	NEB II	300	140	160	8–12	2016–2017	PBDE-47, -153, DP, BDCIPP, DPHP	Caspersen et al. (2019)
SLOVENIA (SI)	SLO CRP	149	82	67	7–10	2018	PBDE-47, -153, −209, DP, HBCDD, DBDPE, BDCIPP, DPHP, BCIPP, BCEP	Stajnko et al. (2020)
SLOVAKIA (SK)	PCB cohort	300	167	133	10–13	2014–2017	BDCIPP, DPHP, BCEP, BCIPP	Hertz-Picciotto et al. (2003)

a Not all studies are nationally representative.

The HBM4EU defined “current exposure” as samples collected between 2014 and 2020 (Gilles et al., 2021), and as such, urine and blood samples from 2136 children aged 6–13 between 2014 and 2021 were used to evaluate current FR exposure in European children (Gilles et al., 2021). Blood and urine (both spot and morning void) samples collected before 2017 were acquired from the biobank of the specific study, while samples from 2017 to 2020 were new collection. Table S1 presents a complete description of the studies, matrices, and institutes involved.

### 2.2 Sample analysis

OPFRs have half-lives of hours to days, as opposed to most halogenated FRs with half-lives of weeks to years (Dvorakova et al., 2021). Due to the rapid rate of metabolization and elimination of OPFRs, it is most appropriate to quantify metabolites in urine instead of the parent OPFR compounds. OPFR metabolites were quantified in children's urine, while the persistent brominated and chlorinated flame retardants were quantified in children's blood samples (Table 2).

**Table 2:** Flame retardants and metabolites analyzed.

Persistent FRs (in blood)	Metabolized FRs (in urine) (Parent compound/metabolite)
PBDE-47, 153, −209 syn-DP, anti-DP α-HBCDD, γ-HBCDD DBDPE BEH-TEBP TBBPA	tris(2-chloroethyl) phosphate (TCEP)/Bis(2-chloroethyl) phosphate (BCEP) tris(1,3-dichloroisopropyl) phosphate (TDCIPP)/Bis(1,3-dicholoro-2-propyl) phosphate (BDCIPP) tris(1-chloro-2-propyl) phosphate (TCIPP)/Bis(1-chloro-2-propyl) phosphate (BCIPP) triphenyl phosphate (TPHP)/Diphenyl phosphate (DPHP)^a^

a DPHP can be a product by itself, a primary metabolite of TPHP, or a secondary metabolite of several OPFRs (Liu et al., 2021).

Eighty-six laboratories from 26 countries were invited to participate in HBM4EU proficiency tests. Seventy-four of these laboratories successfully quantified at least one biomarker and were selected to participate in three rounds of interlaboratory comparison investigations (Esteban López et al., 2021). In this study, all laboratories responsible for the analysis of FR biomarkers in urine and/or blood successfully completed the interlaboratory comparison investigations and external quality assurance schemes, described in detail by Dvorakova et al. (2021).

Six HBM4EU qualified laboratories were involved in the FR analyses. The institutes that participated in the analyses are: the University of Chemistry and Technology, Prague (Czech Republic), Department of Environment and Health, Vrije Universiteit Amsterdam (Netherlands), RECETOX, Masaryk University (Czech Republic), Norwegian Institute of Public Health (Norway), Toxicological Center, University of Antwerp (Belgium), and Friedrich-Alexander-Universität Erlangen-Nürnberg (Germany). The analytical procedures used by the laboratories are presented in the Supplementary Information (Supplementary Table S1 and Text S1). All participating studies in the HBM4EU survey adhered to national and European ethics regulations (Gilles et al., 2021). The ethical information of all participating studies is provided in Table S2 (Gilles et al., 2022). Three laboratories analyzed brominated FRs and DPs using gas chromatography-tandem mass spectrometry (GC-MS/MS). Four laboratories analyzed OPFR metabolites using liquid chromatography-tandem mass spectrometry (LC-MS/MS), and one laboratory used GC-MS. Limits of quantification (LOQs) are given in Tables S3 and S4. With the stringent interlaboratory comparison described above, the concentrations of FRs were deemed comparable, despite differences in analytical methods across laboratories.

### 2.3 Data analyses

In cases where less than 20% of individuals were represented by left-censored data (below limits of detection/quantification), imputation was performed to replace censored data. Imputation can provide more realistic distributions of data than those relying on simple substitution of a fixed value, e.g., 0.5*LOQ (Bernhardt et al., 2015), and was selected for use in the aligned studies of HBM4EU (Govarts et al., 2021). The maximum-likelihood estimation (MLE) method was used to find a distribution best fitting the non-censored data (R Core Team, 2019). From an infinite set of all theoretical log-normal distributions, the MLE method identifies the one for which the probability that given set of values comes from that distribution is maximal. This is done by maximizing a likelihood function of that distribution by computing first derivative of that function. Based on that distribution, random values were generated for the intervals from 0 to LOD (limit of detection) and from LOD to LOQ (limit of quantification).

After imputation, the urinary data were standardized for creatinine, by dividing metabolite concentrations by the known creatinine concentration, while blood serum/plasma data were standardized for lipid content according to the harmonized level of lipid enzymes calculated as Total lipids (mg/dL) = 2.27* (Total cholesterol) + triglycerides + 62.3 mg/dL (Bernert et al., 2007)

A set of univariate analyses were conducted to check the distribution of both the continuous and discrete (categorical) exposure determinants, typically lifestyle factors. If an exposure determinant was not available for more than 40% of the individuals with a known concentration of a compound, the determinant was excluded from further analyses. Discrete exposure determinants with only one factor level, e.g. when all respondents answered “no” to a particular question, or exposure determinants linearly dependent on other determinants were also excluded from the subsequent steps.

Differences between countries were tested using Kruskal-Wallis one-way analysis of variance on the log-transformed concentration data. Due to the sparse exposure determinants matrix, data were only adjusted for sex and age of the participants using the mean value as the reference, and the investigation of exposure determinants was reserved for within-country analyses only.

Finally, a single effect linear regression was conducted on log-transformed data taking all non-excluded exposure determinants using least squares linear regression (Table S7) based on both the variance inflation factor (VIF) and the determinant p-value (Govarts et al., 2021). Exposure determinants with VIF ≤10 or p-value ≤ 0.05 were considered as significantly associated with the flame retardant concentrations. Continuous exposure determinants were investigated using Spearman correlation tests and visualized using scatterplots with 95% confidence level.

### 2.4 Study limitations

The sampling and analytical procedures were aligned according to the HBM4EU standards. However, the aligned study designs were not identical. Each country had different sample sizes (studies ranged from 55 to 413 participants) and prioritized different compounds, as well as different timing of sample collection. Although the sampling years slightly differed, most overlapped and all were within a five-year range (Table 1). Six of the studies provided newly collected samples, while the remainded relied on biobanked samples that had been stored at stable conditions (−80 °C). The difference in storage time is not expected to impact comparability, as storage time has not been found to impact levels of the reported OPFRs in urine (Carignan et al., 2017).

All countries except FR and DE had 100% participants with a European country of birth; DE had 99.3% and FR 95% European birth country representation. In FR, 18 participants were not born in Europe. However, the results of the study are still deemed to be indicative of the European population. The age range of children participating in the study differed (Table 1), and some studies (e.g., CELSPAC (CZ), PCB cohort (SK)) had children on the upper end of the age bracket (10–13 years), while others (e.g., OCC (DK), 3xG (BE)) covered only the lower end of the age bracket (7–8 years). Urine was collected as both morning void and spot samples. In the NEB II study (NO) analyzed BFRs and DP were analyzed in blood plasma, while in the other cohorts they were analyzed blood serum (Table S1). The questionnaire regarding lifestyle factors also differed between countries. Because most lifestyle factors were reported by all countries, or were incompletely reported, multivariate analyses could not be conducted across the full dataset. Although the laboratories participated in a complete QA/QC programme to ensure the quality and comparability of the analytical results (Dvorakova et al., 2021), they had different LODs and LOQs (Tables S3 and S4). However, all laboratories passed the two-stage evaluation process (Dvorakova et al., 2021; Esteban López et al., 2021) and were declared proficient by the HBM4EU project.

## 3 Results and discussions

### 3.1 FR concentrations in children's blood from different European countries

The concentrations of halogenated FRs in children's blood are presented in Table 3. The detection frequencies of all halogenated FR quantified in children's blood are presented in Table S5. Only compounds with detection frequency >40% were considered for statistical analyses. α-HBCDD, γ-HBCDD, and DBDPE were analyzed in blood samples from France, Greece, and Slovenia, but were not present at more than 40% detection frequency (Table S5). BEH-TEBP and TBBPA were only investigated in blood from France but were not present above quantifiable concentrations in any sample (Table S5).

**Table 3:** Median concentrations and 95th percentiles (in parentheses) of halogenated flame retardants in children's blood (ng/g lipid).

Country	PBDE-47	PBDE-153	PBDE-209	ƩDP
France	0.36 (2.81)	35% DF (1.50)	8.73 (159)	34% DF (1.29)
Greece	0.03 (0.43)	0.09 (0.82)	0% DF	2% DF
Slovenia	a15% DF (0.46)	0.29 (1.02)	8% DF (10.10)	16.0 (24.8)
Norway	0.34 (0.93)	19% DF (0.1)	b-	3.17 (33.1)
**All countries**	c**0.32 (1.62)**	**40% DF (0.99)**	**38% DF (115)**	**2.13 (23.6)**

a Where a compound had <40% detection frequency (DF), the DF and 95th percentiles are reported.

b A dash (-) indicates that the compound was not analyzed in blood from that country.

c Values in bold indicate the median and 95th percentile of all data.

**Fig. 1:**
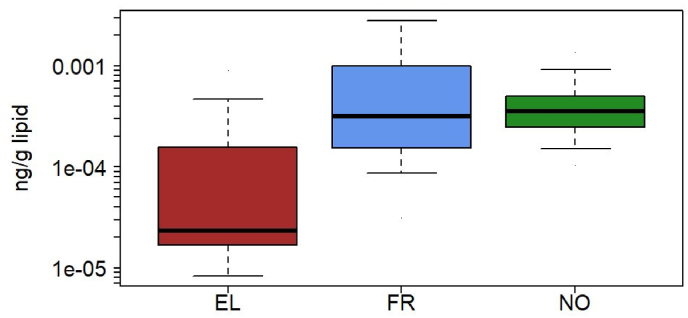
Box plot of lipid-adjusted concentrations of BDE-47 for France (FR), Greece (EL) and Norway (NO). The box indicates median, 25% and 75% percentiles. Whiskers indicate 5% and 95% percentiles.

**Fig. 2:**
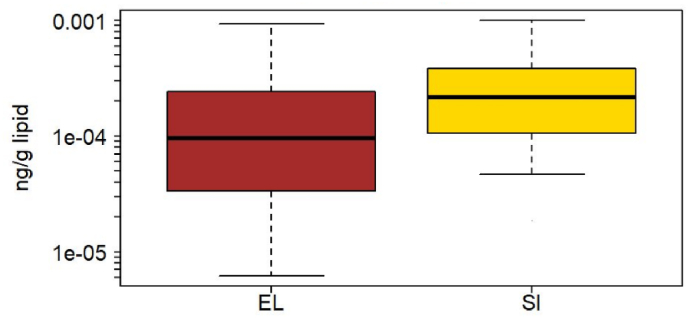
Box plot of lipid-adjusted concentrations of BDE-153 in blood of children from Greece (EL) and Slovenia (SI). The box indicates median, 25% and 75% percentiles. Whiskers indicate 5% and 95% percentiles.

PBDE-47 was present at quantifiable concentrations in France, Greece, Norway, and Slovenia. Slovenia had a 15% detection frequency of BDE-47 and is not included in the inter-country comparison, although we note that the 95th percentile is similar to that of Greece, the other country from the Southern European region (Table 3). When comparing levels across the three countries, Greece had significantly lower concentrations of BDE-47 than either France or Norway (Kruskal-Wallis test, p < 0.001, Fig. 1), in both the unadjusted data as well as in data adjusted for age and sex. BDE-153 was detected in more than 40% of the blood samples from Greece and Slovenia (Fig. 2). The compound was analyzed in blood from France and Norway as well but was detected in only 35% and 19% of the samples, respectively. Greece had significantly lower concentrations of BDE-153 than Slovenia (p < 0.001), both in the adjusted and unadjusted data. Lipophilic compounds such as halogenated FRs are typically associated with dietary exposure and breastfeeding (Pan et al., 2020; Poma et al., 2017). Norway is one of the countries with the highest breastfeeding rate for the first six months of infancy (Theurich et al., 2019), while Greece is known to have low exclusive breastfeeding (Pechlivani et al., 2005), which could be reflected in the concentration differences of PBDEs (Fig. 1, Fig. 2).

BDE-209 was only present above 40% detection frequency in France, although it was analyzed in blood from Greece and Slovenia as well. Quantification limits for BDE-209 for Greece and Slovenia were comparable to or lower than those for France (Table S3), suggesting that the differences in detection are due to population exposures rather than analytical differences. BDE-209 was found at the highest concentration for a single compound in blood compared with all quantified FRs in our study (8.73 ng/g lipid), as expected based on the timing of restrictions and relatively higher recent use of BDE-209 (Abbasi et al., 2019). BDE-47 and BDE-153 were banned from production and use by the Stockholm Convention in 2004, while BDE-209 was only included in the Stockholm Convention in 2017 (Sharkey et al., 2020).

BDE-209 is a compound that can be difficult to quantify due to its high molecular mass (Pietroń and Małagocki, 2017; Stapleton, 2006) and often has low detection frequencies in other studies, typically because of high limits of detections and issues with blank contamination (Crosse et al., 2013; Darrow et al., 2017; Johnson et al., 2010; Wu et al., 2007). In our study, three countries analyzed BDE-209, but the compound was only quantifiable above detection limits in blood from France. Although Dvorakova et al. (2021) stated that Europe has ample laboratories with the capacity to quantify BDE-209, the limitation may be a disconnect between detection and quantification limits and concentrations in biological matrices. DBDPE, which is often considered a replacement for BDE-209, suffers from even more serious analytical challenges due to poorer instrument sensitivity and higher LOQs (Melymuk et al., 2015).

**Fig. 3:**
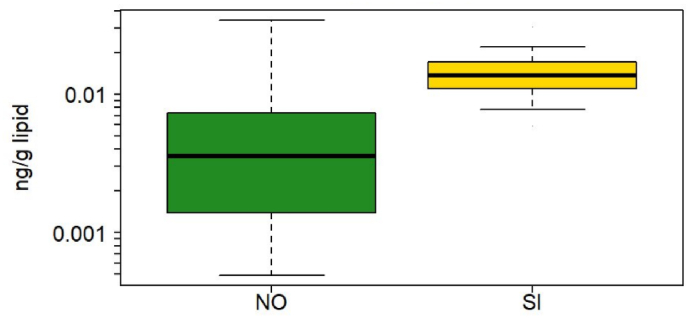
Box plot of lipid adjusted concentrations of ƩDechlorane Plus (DP) in blood of children from Norway (NO) and Slovenia (SI). The box indicates median, 25% and 75% percentiles. Whiskers indicate 5% and 95% percentiles.

Both *syn-* and *anti-*DP congeners were analyzed in blood from France, Slovenia, Greece, and Norway. Blood from France had detection frequencies below 40%. Only one blood sample from Greece had detectable concentrations of *syn-*DP and no *anti-DP* was detected. Slovenia had a median ƩDP concentration of 16 ng/g lipid, which was the highest concentration quantified in our study (Fig. 3). *Anti*-DP was present in 41% of the Slovenian blood samples, and *syn-*DP in only 12%. Norway had 100% and 99% detection frequencies for *syn-* and *anti-*DP, respectively. The median concentration of ƩDP was 3.17 ng/g lipid in blood from Norway, which was significantly lower than concentrations in children's blood from Slovenia (p < 0.001), both for adjusted and unadjusted data. Other countries may also have elevated DP concentrations, but it is difficult to determine due to high LODs (Table S3).

The French ESTEBAN study presented a unique profile in BDE-209 and DP compared with other studies. While detection frequencies are limited for both of these compounds, the comparison of medians, (where available) and 95th percentiles suggest higher exposure to BDE-209 in France and lower exposure to DP compared with Slovenia and Norway. DP has been suggested to be used as a replacement for BDE-209 (Barón et al., 2016) and the dominance of BDE-209 in France compared to DP in other regions may suggest countries are at different points in the transition away from BDE-209. We also note that the French data were among the earliest collected out of all studies, which may be an additional reason for the dominance of BDE-209.

### 3.2 OPFR metabolite concentrations in children's urine from different European countries

The detection frequencies of OPFR metabolites in children's urine are presented in Table S6. The concentrations of OPFR metabolites in children's urine are presented in Table 4.

**Table 4:** Median concentrations and 95th percentiles (in parentheses) of OPFR metabolites in children's urine (μg/g creatinine).

Country	BCEP	BCIPP	BDCIPP	DPHP
Belgium	-a	14% DF (1.02)^b^	0.38 (3.11)	2.41 (7.63)
Czech Republic	0.22 (5.60)	0.10 (0.96)	0.26 (1.95)	2.06 (5.97)
Germany	0.14 (0.86)	0.12 (0.61)	0.64 (2.49)	1.66 (6.21)
Denmark	–	7% DF (1.35)	0.49 (3.71)	1.43 (5.16)
France	–	31% DF (5.04)	0.58 (4.55)	1.92 (9.70)
Slovenia	20% DF (1.04)	18% DF (0.25)	0.64 (1.59)	2.43 (5.47)
Slovakia	20% DF (0.98)	29% DF (0.28)	37% DF (1.16)	2.21 (6.48)
Norway	–	–	17% DF (0.56)	1.78 (7.29)
**All countries**	c**0.23 (1.32)**	**0.19 (1.59)**	**0.38 (2.49)**	**1.91 (6.87)**

a A dash (-) indicates that the compound was not analyzed in urine from that country.

b Where a compound had <40% detection frequency (DF), the DF and 95th percentiles are reported.

c Values in bold indicate the median and 95th percentile of all data.

**Fig. 4:**
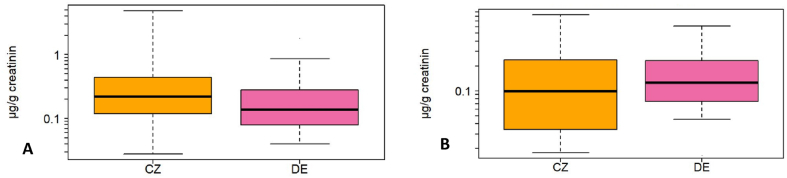
Box plot of creatinine-adjusted concentrations (μg/g creatinine) of (A) BCEP and (B) BCIPP for Czech Republic (CZ) and Germany (DE). The box indicates median, 25% and 75% percentiles. Whiskers indicate 5% and 95% percentiles.

BCEP was analyzed in four countries, but was only present above the LOQ in >40% of urine samples from Germany and Czech Republic (Table 4). Czech samples had significantly higher concentrations of BCEP than German (Kruskal-Wallis; p < 0.001; Fig. 4A), both in unadjusted data and data adjusted for age and sex. BCIPP was analyzed in seven countries, but, as with BCEP, only samples from Germany and Czech Republic had more than 40% >LOQ. In contrast to BCEP, samples from Germany had higher levels of BCIPP than those from the Czech Republic (Kruskal-Wallis; p = 0.019, Fig. 4B), both in unadjusted and adjusted data.

**Fig. 5:**
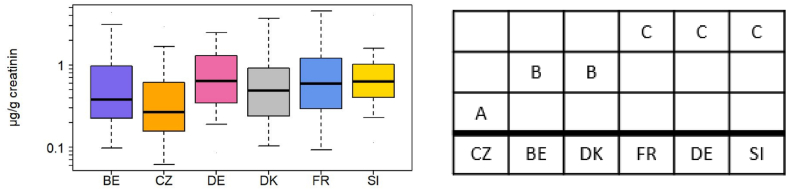
a. Box plot of creatinine adjusted concentrations (μg/g creatinine) of BDCIPP for Belgium (BE), Czech Republic (CZ), Germany (DE), Denmark (DK), France (FR), and Slovenia (SI). The box indicates median, 25% and 75% percentiles. Whiskers indicate 5% and 95% percentiles. **5b.** Mann-Whitney pairwise comparisons between countries. A, B, and C indicate groups of countries with different BDCIPP levels.

BDCIPP was quantified in Belgium, France, Germany, Slovenia, Czech Republic, Norway, Slovakia, and Denmark (Table 4), but was below LOQ in >60% of samples from Slovakia and Norway. A non-parametric Mann-Whitney pairwise comparison revealed three groupings within the countries (Fig. 5). Czech samples had lower concentrations (p < 0.001; Mann-Whitney) than all other countries, while France, Germany, and Slovenia had significantly higher concentrations than the other countries (Fig. 5b). The differences between the country groupings were significant both in the unadjusted data and data adjusted for age and sex. When grouping countries by geographic region (e.g., north/south/east/west according to the UN Geoscheme for Europe) we did not see significant regional differences, suggesting that differences between countries are not due to broad geographic trends (e.g., east-west differences within Europe).

BCEP, BCIPP, and BDCIPP are direct metabolites of chlorinated OPFRs (Liu et al., 2021). The primary compounds (TCEP, TCIPP, and TDCIPP) are usually present in consumer products such as furniture, products containing PUF foam, and electronics (Hoffman et al., 2017). These compounds have been frequently reported in matrices of direct relevance for human exposure, particularly indoor settled dust (Liu and Folk, 2021), indoor air (Yadav et al., 2017), and food products (Blum et al., 2019). While regional differences have been identified in OPFR levels in other matrices, the differences are typically pronounced between Europe, Asia, and North America (He et al., 2015). All countries involved in our study are members of the European Economic Area, which has a common market system enabling the free movement of goods and generally harmonized chemical regulations. Thus, only small differences between countries are to be expected, and these are more likely to be due to small differences in the country's study populations and lifestyle differences between European regions (Section 3.3). The ubiquity of OPFR metabolite contamination can be ascribed to the broad use of chlorinated OPFRs, particularly after the ban on legacy FRs (Percy et al., 2020).

**Fig. 6:**
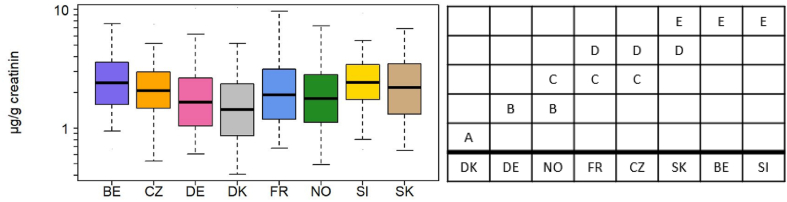
a. Box plot of creatinine-adjusted concentrations of DPHP for Belgium (BE), Czech Republic (CZ), Germany (DE), Denmark, France (FR), Norway (NO), Slovenia (SI), and Slovakia (SK). The box indicates median, 25% and 75% percentiles. Whiskers indicate 5% and 95% percentiles. **6b.** Mann-Whitney pairwise comparisons between countries. Letters groupings indicate groups of countries with different DPHP levels. Countries that do not overlap can be considered to be different from each other.

DPHP was the most widely detected FR, present in 99% of urine samples. Mann-Whitney pairwise comparisons revealed five overlapping groups of countries (Fig. 6b). The country groups were identical in both unadjusted data and adjusted data. Denmark had lower DPHP concentrations than all other countries, while Belgium and Slovenia had the highest (Fig. 6a and b). DPHP was also the OPFR metabolite found at the highest concentration in all countries. In some cases, DPHP concentration in children's urine was an order of magnitude higher than other OPFR metabolites (Table 4). DPHP from Slovenian samples had the highest median concentration of all the OPFR metabolites (2.43 μg/g creatinine). Other studies have also reported DPHP to be ubiquitous in human populations (Butt et al., 2014; Dodson et al., 2014; Hoffman et al., 2017). Unlike the other three quantified OPFR metabolites, DPHP is a non-specific metabolite. It is a metabolite of TPHP, a compound used as a flame retardant and plasticizer, and that is present in a large variety of products including PUF, hydraulic fluid, polyvinyl chloride (PVC), and cosmetic products such as nail polish (Preston et al., 2017). DPHP is also a metabolite of, among others, 2-ethylhexyldiphenyl phosphate (EHDPHP) and resorcinol bis(diphenyl) phosphate (RDP) (Hou et al., 2016). Beyond being a FR, DPHP itself is also produced as a plasticizer, and used in certain chemical reagents and intermediate of pesticides, medicines, and organic material, is a polymerization catalyst and is an additive in paints and coatings (Liu et al., 2021). Considering the multiple exposure sources, it is not surprising that DPHP was the compound found most frequently at the highest detection frequencies in most countries.

### 3.3 Comparisons with other studies

Only a limited number of studies have investigated FRs in children between the ages of 6 and 12 years old. Many studies have quantified FRs in infants or toddlers (often in conjunction with samples from the mother) (Caspersen et al., 2016; He et al., 2018a; Morello-Frosch et al., 2016) or in adults (Brasseur et al., 2014; Lenters et al., 2013; Sochorová et al., 2017; Wang et al., 2019). However, since Moya et al. (2004) noted significant differences in both behavior and physiology between different age groups, we do not expect similar concentrations of FRs across age groups. Typically, when a study includes both adults and children, children have higher concentrations of BFRs than adults (Fischer et al., 2006; Toms et al., 2009; van den Eede et al., 2015). One study that quantified PBDEs in four members of the same household found that the children had 2- to 5-fold higher concentrations than the adults despite identical living conditions and diets (Fischer et al., 2006).

BDE-47 and 153 in children's blood from our study were both comparable, if slightly lower than studies from China (Table S8). The HELIX study analyzed chemicals, including BDE-47 and 153, in children and mothers from various European countries, providing much overlap with our study (Haug et al., 2018). The concentrations found in the HELIX study were comparable to our study. Only BDE-47 from France (0.36 ng/g lipid) was slightly higher in our study than in the HELIX study (0.27 ng/g lipid); the other concentrations from Greece and Norway were slightly higher in the HELIX study (Haug et al., 2018). Studies from the US and Canada reported concentrations of BDE-47 and -153 that were two orders of magnitude higher than what was found in our study (Table S8). PBDEs are typically significantly higher in studies from the US, and Canada to a lesser extent, when compared with other global regions (Frederiksen et al., 2009; Siddiqi et al., 2003; Sjödin et al., 2008) due to higher past use of PBDEs. Serum from Norway (Thomsen et al., 2007) had higher concentrations than what was found in Norway by the NEB II study. However, it should be kept in mind that the samples were collected in 1998, and it is known that the use of, and consequently exposure to, PBDEs has decreased since the 1990s. It was unexpected to see that BDE-209 from France in our study (8.73 ng/g lipid) was the second-highest concentration of this comparison, after one study from China (95 ng/g lipid (Guo et al., 2016)).

Previous studies have analyzed BFRs in adults from European countries. In 2015, BFRs were quantified in serum from 300 Czech adults. PBDEs were only detected in 9% of the samples above detection limits (Sochorová et al., 2017). Other studies quantified PBDEs in serum from France (Brasseur et al., 2014), Poland, Ukraine (Lenters et al., 2013), and other European countries (Garí and Grimalt, 2013). Almost all concentrations from prior studies in adults were higher than the concentrations found in our study, which we attribute to the more recent sample collection. All children that were involved in our study were born between 2001 and 2015. The European Union restricted the use of Penta- and Octa-BDE commercial products from 2004, and the lower brominated congeners of PBDEs (tetra-, penta-, hexa-, and heptabromodiphenyl ethers) were listed for elimination under Annex A of the Stockholm Convention on Persistent Organic Pollutants in 2009 (Stockholm Convention, 2019). Though these literature-based comparisons should be interpreted with caution, the fact that PBDE concentrations in children's blood from our study are at comparable and lower concentrations than adults from studies in 1990s and early 2000s can be an indication that regulation restricting PBDE production and use are effective at reducing exposure.

While BFRs are reported in lipid-standardized units (e.g., ng/g lipid mass) enabling comparison across studies, studies that quantified OPFRs in urine do not have a standardized unit of quantification; data are presented in unadjusted units (e.g, μg/L), specific gravity (SG) normalized, or creatinine-standardized (μg/g crt), as is the case for our data. Given the limited data on OPFR metabolites in children, we have included studies using unadjusted of SG normalized urine concentrations in our comparison, and done an average-based recalculation based on ratios of creatinine to specific gravity for this age group of children (Table S8). Given the use of this recalculation, the comparisons with other studies should be interpreted with caution.

All BCEP and BCIPP concentrations from our study and previous studies were within the same order of magnitude. BDCIPP concentrations in urine were an order of magnitude higher in US compared with China; and European values from our study fell between these two levels (Table S8). DPHP concentrations were similar in Europe and the US, but China and Japan had lower concentrations of the compound in children's urine (Table S8).

### 3.4 Lifestyle factors associated with flame retardant exposure

Forty lifestyle factors were examined and the responses recorded by country, presented in Table S9.

A significant inverse correlation between age and FR exposure was observed for both BDCIPP and DPHP in France (p < 0.001 and p = 0.005) and Germany (p = 0.002 and p = 0.006) (Table S9). This agrees with the findings from several other studies, where OPFR concentrations decreased with children's age (Chen et al., 2018; He et al., 2018a; Sun et al., 2018; van den Eede et al., 2015; Zhang et al., 2018)*,* and is likely related to more hand-to-mouth activity in younger children, resulting in increased exposure to dust-containing FRs (Cequier et al., 2015; Ionas et al., 2016; Moya et al., 2004).

In addition to age, our study found associations between FR concentrations and the physical structure of the indoor environment, cleaning habits, socioeconomic determinants, and diet (Table S9). Cequier et al. (2015) noted that the residential environment might be a more important exposure pathway of FRs than food, which agrees with the findings of our study. Renovation status, PVC floor, and vacuum and cleaning frequency per week were associated with FR concentrations in children from several countries (Table S9). In the Czech Republic, BCIPP (p = 0.012) was higher in houses that have been renovated within two years before the study, compared with unrenovated homes (Table S9). BCIPP was also higher in newer homes in France (p = 0.007), where home age correlated negatively with BCIPP concentrations. BDCIPP was lower in Czech children from homes with PVC floors (p = 0.013). Similarly, lower BDE-153 was quantified in children's blood from Slovenian households with PVC walls than in the blood from homes without (p = 0.036). The reason for this is unknown.

BDCIPP and DPHP were both higher in Czech households that “never” or “rarely” clean (p = 0.003 and p < 0.001). Similarly, BDE-47 was higher in Greek households that “rarely” vacuum (p = 0.009) and BDE-153 in Slovenian households that “never” or “rarely” clean (p = 0.027) (Table S9). Since FRs are present in indoor house dust (Ali et al., 2012; Fromme et al., 2014; Jílková et al., 2018), removing dust from living spaces is a good way to limit inhalation and dermal exposure, and has been linked to lower levels of FRs in indoor dust (Sugeng et al., 2018). However, BDE-47 was higher in French households that “often” vacuum (p = 0.009). The reason for this disparity is unknown.

The only significant association between measured FR levels and consumption of food in our study was observed for DPHP in seafood from Belgium (p = 0.021). Dietary exposure to OPFRs has been indicated in prior studies (He et al., 2018b; Xu et al., 2017), but was not very prominent in our study Similarly, the effect of passive smoking was almost negligible in this study; only BDCIPP in Slovenia was significantly higher in children from smoking households (p = 0.047).

In the French and Belgium studies, BDCIPP concentrations were positively correlated with time spent in a car (ρ = 0.189, p = 0.001 for France and Belgium ρ = 0.195, p = 0.024). TDCIPP, the parent compound of BDCIPP, is known to be present in car upholstery, where elevated temperatures can lead to an increase in volatilization (Phillips et al., 2018). There is evidence from the USA that longer commutes lead to increased TDCIPP exposure (Reddam et al., 2020).

An interesting feature was observed when studying socioeconomic factors. BCIPP (p = 0.006), and DPHP (p = 0.002) were higher in German households where the father was employed, versus households where the father was unemployed. Another socioeconomic association was found with household education in Germany, where households with the lowest levels of education had the lowest levels of DPHP in children's urine (p = 0.005). Conversely, the legacy FR BDE-47, was prominent in households where the father was unemployed (p = 0.016), as seen in blood from Norway (Table S9). This suggests a link between socioeconomic status and furniture or product replacement rates. We hypothesize that the higher levels of BDE-47 in houses where the father is unemployed are linked with lower income and related to the older furnishing or appliances containing legacy FRs (e.g., purchased before restrictions on PBDEs). In contrast, employment is linked to higher-income households that have higher purchasing power and are more likely to replace products and furnishings, leading to more products containing OPFRs instead of the now-banned PBDEs. Previous studies have linked BCIPP exposure with the number of electrical appliances and electronics in a home (He et al., 2018a; Sun et al., 2018), which can also be an indication of socioeconomic position.

Gender appeared to impact concentrations in a few instances. Slovenian boys had significantly higher BDCIPP and BDE-153 than girls (p = 0.021 and p = 0.01). Similarly, boys had significantly higher concentrations of BCIPP in Czechia (p = 0.019) and BDE-47 in Greece (p = 0.024). However, since this trend was not consistent throughout the study, the effect of gender on FR exposures should be interpreted with caution.

Most associations between FR concentrations and lifestyle factors were found for BDCIPP and DPHP. These compounds had the highest detection frequencies of the studied compounds. The information gained should be interpreted with caution. A more in-depth study of the lifestyle factors correlating with FR and other chemical compounds is recommended.

## 4 Conclusions

Halogenated FRs were quantified in children's blood from four countries and OPFRs in urine from children from eight countries. This was the largest aligned study across multiple European countries to quantify FRs in children. This was also the first large-scale comparative study of OPFRs between different countries, providing valuable data to both researchers and policymakers. OPFR metabolites, particularly BDCIPP and DPHP, have ubiquitous distribution in Europe, with limited differences between countries, perhaps due to the open market conditions. OPFR concentrations should be critically evaluated by regulatory institutions due to their high prevalence and indications of endocrine-disrupting effects. The concentrations of BDE-47 in children's blood collected recently were comparable and lower than BDE-47 in adult samples from several years ago, suggesting that the regulation of PBDEs does mitigate the exposure of the compounds to humans. This study has highlighted the need to further build capacity to enable more laboratories to analyze OPFRs, BDE-209 and other halogenated alternative FRs, such as DP, DBDPE, TBBPA, and DBDPE. While it was difficult to ascribe specific lifestyle factors to flame retardant concentrations, factors concerning cleaning, socioeconomic status, and physical properties of the residence had the most significant correlations. It is recommended that future studies further investigate these and other lifestyle factors to better understand FR exposure to children.

## Funding

The HBM4EU project has received funding from the European Union’s Horizon 2020 research and innovation program under grant agreement No 733032 and received co-funding from the author's organizations. Co-funding by German Ministry for the Environment, Nature Conservation, Nuclear Safety and Consumer protection is gratefully acknowledged. The Norwegian Institute of Public Health (NIPH) has contributed to funding of the Norwegian Environmental Biobank (NEB) and laboratory measurements have partly been funded by the 10.13039/501100005416Research Council of Norway through research projects (275903 and 268465). Support was also provided by the Research Infrastructure RECETOX RI (No. LM2018121) and CETOCOEN EXCELLENCE (CZ.02.1.01/0.0/0.0/17_043/0009632), the Operational Programme Research, Development and Innovation – project Cetocoen Plus (CZ.02.1.01/0.0/0.0/15_003/0000469) and the European Union’s Horizon 2020 research and innovation programme under grant agreement No. 857560. The Slovenian SLO-CRP study was co-financed by the Jožef Stefan Institute program P1- 0143, and a national project “Exposure of children and adolescents to selected chemicals through their habitat environment” (grant agreement No. C2715-16-634802). The PCB cohort was supported by 10.13039/100009647Ministry of Health of the Slovak Republic, grant no. 2012/47-SZU-11 and by the Slovak Research and Development Agency, grant no. APVV-0571-12. The Cross-Mediterranean Environment and Health Network (CROME) study has been co-funded by the 10.13039/501100000780European Commission research funds of Horizon 2020.

## Declaration of competing interest

The authors declare that they have no conflict of interest.
