# Supplementary material for: Exposure to flame retardants in European children — Results from the HBM4EU aligned studies
Source: Int J Hyg Environ Health. 2023 Jan;247:114070. doi: 10.1016/j.ijheh.2022.114070 (PMC9758617; doi:10.1016/j.ijheh.2022.114070)
Supplement: Multimedia component 1 [file mmc1.docx]

**Supplementary Information for**

**Exposure to flame retardants in European children — Results from the HBM4EU aligned studies**

Veronica van der Schyff^1^, Jiri Kalina^1^, Eva Govarts^2^, Liese Gilles^2^, Greet Schroeters^2,3^, Argelia Castaño^4^, Marta Esteban-López^4^, Jiri Kohoutek^1^, Petr Kukucka^1^, Adrian Covaci^5^, Gudrun Koppen^2^, Lenka Andryskova^1^, Pavel Piler^1^, Jana Klanova^1^, Tina Kold Jensen^6^, Loic Rambaud^7^, Margaux Riou^7^, Marja Lamoree^8^, Marike Kolossa-Gehring^9^, Nina Vogel^9^, Till Weber^9^, Thomas Göen^10^, Catherine Gabriel^11,12^, Dimosthenis A. Sarigiannis^11,12,13^, Amrit Kaur Sakhi^14^, Line Småstuen Haug^14^, Lubica Palkovicova Murinova^15^, Lucia Fabelova^15^, Janja Snoj Tratnik^16^, Darja Mazej^16^, Lisa Melymuk^1, *^

^1^ RECETOX, Faculty of Science, Masaryk University, Kotlarska 2, Brno, Czech Republic

^2^ VITO Health, Flemish Institute for Technological Research (VITO), Mol, 2400, Belgium

^3^ Department of Biomedical Sciences, University of Antwerp, 2020 Antwerp, Belgium

^4^ National Centre for Environmental Health, Instituto de Salud Carlos III, Majadahonda, Madrid, Spain

^5^ Toxicological Center, University of Antwerp, 2610 Wilrijk, Belgium

^6^ Department of Environmental Medicine, Institute of Public Health, University of Southern Denmark, Odense, 5000, Denmark

^7^ Santé Publique France, French Public Health Agency (ANSP), Saint-Maurice, 94415, France

^8^ Vrije Universiteit, Amsterdam Institute for Life and Environment, Section Chemistry for Environment & Health, De Boelelaan 1108, 1081 HZ Amsterdam, Netherlands

^9^ German Environment Agency (UBA), 06844 Dessau-Roßlau, Germany

^10^ IPASUM - Institute and Outpatient Clinic of Occupational, Social and Environmental Medicine; Henkestrasse 9-11, 91054, Erlangen, Germany

^11^ Environmental Engineering Laboratory, Department of Chemical Engineering, Aristotle University of Thessaloniki, 54124, Thessaloniki, Greece

^12^ HERACLES Research Center on the Exposome and Health, Center for Interdisciplinary Research and Innovation, Balkan Center, Bldg. B, 10th km Thessaloniki-Thermi Road, 57001, Greece

^13^ Environmental Health Engineering, Institute of Advanced Study, Palazzo del Broletto - Piazza Della Vittoria 15, 27100 Pavia, Italy

^14^ Department of Environmental Health, Norwegian Institute of Public Health, Oslo, Norway

^15^ Faculty of Public Health, Slovak Medical University, Bratislava, 833 03, Slovakia

^16^ Department of Environmental Sciences, Jožef Stefan Institute, Ljubljana, 1000, Slovenia

* Corresponding author:

Lisa Melymuk

[lisa.melymuk@recetox.muni.cz](mailto:lisa.melymuk@recetox.muni.cz)

RECETOX, Faculty of Science, Masaryk University, Kotlarska 2, Brno, Czech Republic

**Supplementary Text**

[Analytical procedures of each study S6](#_Toc106352216)

**Tables**

[Table S1 Details on studies and laboratories performing the analyses S3](#_Toc114818585)

[Table S2 Ethics committees and funding information of participating HBM4EU aligned studies S11](#_Toc114818586)

[Table S3 Limits of detection (LOD) and limits of quantification (LOQ) of compounds analyzed in serum/plasma (μg/L). S12](#_Toc114818587)

[Table S4 Limits of detection (LOD) and limits of quantification (LOQ) of compounds analyzed in urine (μg/L). S12](#_Toc114818588)

[Table S5 Detection frequencies of halogenated flame retardants quantified in children’s serum/plasma different countries in this study S13](#_Toc114818589)

[Table S6 Detection frequencies of organophosphate flame retardants quantified in children’s urine different countries in this study S13](#_Toc114818590)

[Table S7 Parameters from single-effect linear regression S14](#_Toc114818591)

[Table S8 Flame retardant concentrations in children from previous studies S15](#_Toc114818592)

[Table S9 Lifestyle factors questionnaire and responses by country S15](#_Toc114818593)

[Table S10 Lifestyle factors with significant association to flame retardant concentrations S20](#_Toc114818594)

Table S1 Details on studies and laboratories performing the analyses

| **Country** | **Study** | **Cohort profile** | **Compound** | **Matrix** | **Sample collection** | **Laboratory** |
| --- | --- | --- | --- | --- | --- | --- |
| Belgium | 3xG | The 3xG study was conducted in three Flemish communities of the province Antwerp. The study was initiated as a birth cohort in 2010-2015 to follow-up health and health determinants in a regional setting. The follow-up examination included a total of 212 participants between 6-8 years, for which a subset of 133 participants contributed to the HBM4EU aligned studies. | BDCIPP  BCIPP  DPHP | Morning urine | New urine samples were collected between January 2019 and March 2020. | Toxicological Center, University of Antwerp |
| Czech Republic | CELSPAC | CELSPAC: School children study (Central European Longitudinal Studies of Parents and Children: Teenagers) was conducted in the South Moravia schools in the Czech Republic between 2019 to 2020. The main aim of this cross-sectional study was to assess the multiple factors potentially affecting the physical fitness of school children. A total sample of 195 school children was recruited to the study, completed questionnaires, underwent examinations and donated urine for the CELSPAC biobank. | BCEP  BCIPP  BDCIPP  DPHP | Spot urine | New collection (2019/2020) | Trace Analytical Laboratory, RECETOX, Masaryk University |
| Germany | GerES V | The German Environmental Survey (GerES) is a representative population study carried out in order to determine the exposure to pollutants of the general population in Germany. GerES V investigated children and adolescents by determining, on a representative basis, the body burden of pollutants and the exposure to pollutants at home  (European Commission, 2021a). | BCEP  BCIPP  BDCIPP  DPHP | Morning urine | Biobanked samples of which 300 were newly analyzed for aligned studies | Institute and Outpatient Clinic of Occupational, Social and Environmental Medicine (IPASUM), Friedrich-Alexander-Universität Erlangen-Nürnberg |
| Denmark | OCC | The OCC study was initiated in the Municipality of Odense in Southern Denmark. Recruitment occurred from 2010-2013. Urine was collected from children involved at 7 year timepoint. | BCIPP  BDCIPP  DPHP | Spot urine | New urine collection from 2018-2019 | Toxicological Center, University of Antwerp |
| France | ESTEBAN | Cross-sectional study in the general population, representative from the French population at national level.  (European Commission, 2021b) | anti-DP  syn-DP  PBDE-47  PBDE-153  PBDE-209  TBBPH  BEH-TEBP  DBDPE  αHBCDD  γHBCDD | Blood serum | Biobanked samples collected in 2014/2016 | Department of Food Analysis and Nutrition (VSCHT), University of Chemistry and Technology, Prague |
|  |  |  | BCIPP  BDCIPP  DPHP | Morning urine | Biobanked samples collected in 2014/2016 | Department Environment & Health, Vrije Universiteit Amsterdam |
| Greece | CROME | The CROME study was initiated as a parent-children cohort in the city of Thessaloniki investigating the levels of environmental pollutants and biochemical indicators of exposure. Participants were invited through bilateral meetings and word of mouth as due to the covid-19 pandemic the initial planning, i.e. to take place through the school structures was not feasible. | anti-DP  syn-DP  PBDE-47  PBDE-153  PBDE-209  DBDPE  αHBCDD  γHBCDD | Blood serum | New serum samples were collected for OPFR metabolites in the frame of CROME cohort between July 2020 and March 2021 | Trace Analytical Laboratory, RECETOX, Masaryk University |
| Norway | NEB II | Cross-sectional study, including children throughout Norway | anti-DP  syn-DP  ƩDP  PBDE-47  PBDE-153 | Blood plasma | Biobanked samples collected in 2016/17 | Environmental Exposure and Epidemiology, Norwegian Institute of Public Health |
|  |  |  | DPHP  BDCIPP | Spot urine | Biobanked samples collected in 2016/17 |  |
| Slovenia | SLO CRP | Cross sectional study conducted in Mura region, Slovenia to assess exposure to selected chemicals in children and adolescents through their living environment. (Stajnko et al., 2020) | anti-DP  syn-DP  PBDE-47  PBDE-153  PBDE-209  αHBCDD  γHBCDD  DBDPE | Blood serum | New serum samples were collected between January and June 2018. | Trace Analytical Laboratory, RECETOX, Masaryk University |
|  |  |  | DPHP  BCEP  BCIPP  BDCIPP | Spot urine | New urine samples were collected between January and June 2018. |  |
| Slovakia | PCB cohort | Longitudinal birth cohort study in region of Eastern Slovakia impacted by past manufacturing of PCBs.  (Hertz-Picciotto et al., 2003) | BCEP  BCIPP  BDCIPP  DPHP | Spot urine | Biobanked samples | Trace Analytical Laboratory, RECETOX, Masaryk University |

# Supplementary text. Analytical procedures of each study

**3xG OPFR metabolites**

Urine samples were analyzed for OPFR metabolites at the Toxicological Center, University of Antwerp, BE using the procedure described by Bastiaensen et al. (2018). Urine samples (1 mL) were spiked with mass-labelled internal standards (5 ng), adjusted to pH 6 with phosphate buffer (1 M) and deconjugated with ß-glucuronidase (2 mg/mL). Samples were extracted on Bond-Elut C18 cartridges (3 mL, 200 mg), conditioned with 3 mL of methanol and 2 mL of water. Target analytes were eluted with methanol (3 mL), concentrated and filtered in a micro-centrifuge (0.2 µm nylon, VWR). Final extracts (water:methanol 1:1) were injected on a Agilent 1290 Infinity liquid chromatography system coupled to a triple quadrupole mass spectrometer (ESI-6460, Agilent). Separation was obtained by a Kinetex Biphenyl column (2.1 mm × 100 mm, 2.6 µm; Phenomenex) and mobile phases of water with 2% methanol (A) and methanol with 2% water (5 mM ammonium acetate as additive). Target analytes were identified through dynamic multiple reaction monitoring (dMRM) in positive and negative ionization. Quantification of target analytes was performed with calibration curves in neat solvents ranging from 0.04 to 10 ng/mL, except for BCIPP and 4-HO-DPHP for which the calibration ranged from 0.2 to 50 ng/mL. Procedural blanks were analyzed to check for background contamination. DPHP, DNBP and TCEP were found at trace levels in procedural blanks (0.14, 0.08 and 0.03 ng/mL, respectively). These blank concentrations were subtracted from values in urine samples. Limits of quantification (LOQ) were determined as three times the standard deviation of procedural blank concentrations (for DPHP, DNBP and TCEP) or as the concentration corresponding to a signal-to-noise ratio of 10 in spiked urine samples. Successful participation in inter-laboratory comparison exercises (HBM4EU ICI/EQUAS and OSEQAS, 2018–2020) assured additional external quality control for several target analytes (DPHP, BDCIPP and BCIPP).

**CELSPAC OPFR metabolites**

Urine samples were analyzed at the RECETOX Trace Analytical Laboratories, Brno, CZ. Urine samples were brought to room temperature, homogenized by vortex and 500 µl of each urine sample was transferred to 96-well plate. Samples were spiked with 5 ng of isotopically-labelled internal standards, and 500 µL of 10 mM NH4Ac was added. Target analytes were extracted using 96-well SPE plate Oasis WAX (60mg). Conditioning was done using 2mL 5% NH4OH in MeOH and then 2 mL 10mM NH4Ac per well. Samples were washed using 1mL 30% MeOH (pH=5) and eluted using 2mL 5% NH4OH in MeOH to the 96- well plate containing 10uL DMSO/well as keeper. After evaporation to DMSO under nitrogen stream, samples were diluted by 400 ul 50% MeOH and homogenized. Final extracts were injected on an Agilent 1200 series liquid chromatography (HPLC) system coupled to a tandem mass spectrometer (AB Sciex QTrap 5500) operating in negative electrospray ionization mode. Chromatographic separation was accomplished using a Waters Acquity BEH C-18 analytical column (100 x 2.1 mm, 1.7 μm particle size) maintained at 30°C and equipped with Acquity VanGuard pre-column. The mobile phases for the gradient separation of the analytes were 1.0 mM water solution of ammonium fluoride (component A) and methanol with an addition of 1.0 mM ammonium fluoride (component B), with a flow rate was 0.3 mL·min-1, and the injection volume was 5 μL. Quantification of target analytes was performed by isotope dilution method using 2D-labeled BCEP, BCIPP, BDCIPP and DPHP. The linear quantification range (MRM mode) was 0.1-100 µg/L urine, with limits of quantification from 0.09 to 1.0 µg/L urine (MQL, calculated as 10* the standard deviation (SD) of the blank sample). Successful participation in inter-laboratory comparison exercises (HBM4EU ICI/EQUAS and OSEQAS, 2018–2020) assured additional external quality control for DPHP, BDCIPP and BCIPP.

**GerES V OPFR metabolites**

Urine samples were analyzed by the Institute and Outpatient Clinic of Occupational, Social and Environmental Medicine of the University of Erlangen-Nuremberg. Urine samples (5 mL) were acidified and spiked with an internal standard solution which included stable isotope labeled compounds structurally identical with the analytes (BDCPP-d2, BCEP-d8, DPhP-d10 and BDCPP-d10). The urinary solutions were extracted by SPE using ENV+ cartridges. After elution the extract were evaporated to dryness and the residues underwent a derivatization with pentaflurobenzyl bromide at 65 °C for 16 hours. Final extracts were injected on an Agilent 7000A gas chromatography (tandem mass spectrometer (GC-MS/MS) using electron impact ionization for DPhP, BCEP and BCPP and an Agilent 5975D gas chromatography-mass spectrometry (GC-MS) system using negative chemical ionization for BDCPP. The procedure was calibrated by standard solution prepared in pooled urine. The quality was controlled by analyzing samples of quality control materials in each series and the application of control charts. Successful participation in inter-laboratory comparison exercises (HBM4EU ICI/EQUAS 2018–2020) assured additional external quality control for DPHP, BDCIPP, BCEP and BCIPP.

**OCC OPFR metabolites**

Same as 3xG

**ESTEBAN OPFR metabolites**

Urine samples were analyzed by the Vrije Universiteit Amsterdam. Deuterated isotopically labeled internal standard (50 µl, 50 ng/ml DPHP-d10, BCIPP-d12 and BDCIPP-d10, all Toronto Research Chemicals) was added to 0.5ml urine. The conjugated metabolites were hydrolyzed by 5 µl β–glucuronidase (0.7 U per sample, Roche) under 0.5 M acetate buffered conditions at pH 6.2. The extraction and separation were done using an online SPE-LC configuration (Elute, Bruker). The extracts were trapped on a C18 cartridge (Waters Xbridge, 4.6x20mm, 5µm) and washed with 1 ml 0.2% acetic acid (v/v). The analytes were eluted from the cartridge to the analytical column (Waters Xbridge C18 100x2.1mm, 2.5µm) by a gradient of 10 mM ammonium acetate and methanol, starting at 5% methanol for 1 m, followed by a linear increase in 5 m to 95% methanol and followed by isocratic elution under these conditions for 5 m. The target compounds were detected with a mass selective detector (MS/MS, EVOQ, Bruker) using electrospray ionization operated in the negative ion mode (DPHP: 249 -> 93/155, BCIPP: 319 -> 35 & 317 -> 35, BDCIPP: 249 -> 35 & 251 -> 35).

Successful participation in inter-laboratory comparison exercises (HBM4EU ICI/EQUAS and OSEQAS, 2018–2020) assured additional external quality control for DPHP and BDCIPP. Background contaminations were checked by analyzing procedure and solvent blanks.

**ESTEBAN FRs in serum**

Serum samples were analyzed by the Department of Food Analysis and Nutrition from University of Chemistry and Technology, Prague (UCT Prague), following the method published in Svarcova et al., 2019. The analytical method is based on the simultaneous determination of non-polar (BDE 47, BDE 153, BDE 209, DBDPE, syn-DP, anti-DP) and less non-polar compounds (HBCDD, TBBPA, 2,4,6-TBP) in serum. To each sample (3,5 ml minimum) surrogate standards were added, specifically a mixture of 13C-HBCDD isomers, 13C-TBBPA, BDE 37, BDE 77 and 13C-BDE 209. Non-polar compounds were extracted by three-step solvent extraction using a mixture of n-hexane:diethylether (9:1, v/v), followed by purification using a solid-phase extraction (SPE) on a Florisil® column. HBCDD, TBBPA and 2,4,6-TBP were further extracted after the first step using a modified QuEChERS method. Depending on the polarity and volatility of target compounds, a different methods were used for identification and quantification of compounds. Gas chromatography coupled to (tandem) mass spectrometry (GC–MS/(MS)) was used for BDEs, DP-syn, DP-anti and DBDPE, and ultra-high performance liquid chromatography coupled to triple quadrupole tandem mass spectrometry (UHPLC–MS/MS) was used for HBCDD, TBBPA and 2,4,6-TBP. Successful participation in inter-laboratory comparison exercises (HBM4EU ICI/EQUAS and OSEQAS, 2018–2020) assured additional external quality control all serum analyses. For further details, please see the published study by Svarcova et al. (2019).

**CROME FRs in serum**

Serum samples were analyzed at the RECETOX Trace Analytical Laboratories, Brno, CZ. Thawed blood sera were extracted using liquid-liquid extraction (LLE). 250 µL of serum was spiked with 40 µL internal standard mix (13C labelled compounds) in isopropanol (IPA). A further 250 µL ethanol was added for protein precipitation and about 1 g of activated silica to form a slurry. Sera were extracted three times using 5 mL of dichloromethane/n-hexane (1:4, v/v) mix. The extracts were reduced in volume under stream of nitrogen. Clean-up was performed using open column (1 cm i.d.) silica chromatography. The column was filled (bottom to top) with 2 cm of activated silica, 3 cm H2SO4 modified (44%) silica and 10 cm sodium sulphate. The extracts were quantitatively loaded on the column and eluted using 30 mL dichloromethane/n-hexane (1:1 v/v). The eluates were reduced in volume under stream of nitrogen using SuperVap (FMS, USA) apparatus, then transferred into conical GC vial, volume further reduced to 15 µL (in nonane) under nitrogen stream and 20 µL syringe standards were added. Gas chromatography atmospheric chemical ionization tandem mass spectrometry (GC-APCI-MS/MS) was used for analysis. The GC was fitted with a 15 m x 0.25 mm x 0.10 µm RTX-1614 column (Restek, USA). Injection was splitless 1 μL at 280°C, with He as carrier gas at 1.5 mL min-1. The GC temperature programme was 80°C (1 min hold), then 20°C min-1 to 250°C, followed by 1.5°C min-1 to 260°C (2 min hold) and 25°C min-1 to 325°C (5 min hold). The MS was operated in MRM mode. Data were processed using Waters (UK) TargetLynx Software. Internal QA/QC was accomplished through three procedural blanks, and replicates of SRM 1957 (NIST, USA) and HBM4EU inter-laboratory study test material; blank-based LOD values were used. Successful participation in inter-laboratory comparison exercises (HBM4EU ICI/EQUAS and OSEQAS, 2018–2020) assured additional external quality control all serum analytes.

**NEB II OPFR metabolites**

Urine samples were analyzed by the Environmental Exposure and Epidemiology section, Norwegian Institute for Public Health following the method of Cequier *et al* (2016). Clean up and pre-concentration with solid-phase extraction (Strata X-AW) and analysis using UPLC-QTOF Details about standards, internal standards and exact masses used are as described by *Cequier et al.* (2014).

**NEB II FRs in plasma**

Plasma samples were analyzed by the Environmental Exposure and Epidemiology section, Norwegian Institute for Public Health following the method of Caspersen *et al*. (2016). Solid-phase extraction (Oasis HLB) and clean-up on a sulphuric acid silica column and analysis using GC-MS/MS as described by Frederiksen *et al* (2020).

**SLO CRP OPFR metabolites**

Same as CELSPAC

**SLO CRP FRs in serum**

Serum samples were analyzed at the RECETOX Trace Analytical Laboratories, Brno, CZ (Palát et al., 2022). Samples (200 μL) were allowed to stabilize at ambient temperature (~20 °C) for 60 minutes and transferred to 2 mL amber glass vials and fortified with 13C internal standards in 40 μL IPA and vortexed for 20 s. Protein precipitation was performed by addition of 600 μL MeCN. These samples were then extracted by 96-well plate solid phase extraction (SPE; 60 mg Oasis HLB) using DCM:Hex (1:19 (v/v)), preconditioned with 1 mL DCM:Hex (1:19 (v/v)), followed by 1 mL MeOH and finally 1 mL ultrapure water. A Phree plate was prepared by the addition of 0.1 g prebaked anhydrous Na2SO4 to each well and wells prewashed with 1 mL DCM:Hex (1:19 (v/v)). The Oasis HLB plate was then stacked atop the Phree plate, and samples were eluted by addition of 1.2 mL DCM:Hex (1:19 (v/v)). The eluates were evaporated under nitrogen to ~600 μL and transferred into GC vials. After that 20 μL of nonane was added as keeper and extracts were evaporated under nitrogen to ~20 μL volume. Standards 13C-BDE 77 and 13C-BDE 138: 1000 pg, 13C-PCB 162: 500 pg were added and extracts concentrated under nitrogen to final volume of ~40 μL for analysis of FRs. Gas chromatography atmospheric chemical ionization tandem mass spectrometry (GC-APCI-MS/MS) was used for analysis. The GC was fitted with a 15 m x 0.25 mm x 0.10 µm RTX-1614 column (Restek, USA). Injection was splitless 1 μL at 280°C, with He as carrier gas at 1.5 mL min-1. The GC temperature programme was 80°C (1 min hold), then 20°C min-1 to 250°C, followed by 1.5°C min-1 to 260°C (2 min hold) and 25°C min-1 to 325°C (5 min hold). The MS was operated in MRM mode. Data were processed using Waters (UK) TargetLynx Software. Internal quality controls was accomplished through processing six solvent blanks and ten replicates of SRM 1957 and SRM 1958 serum. Successful participation in inter-laboratory comparison exercises (HBM4EU ICI/EQUAS and OSEQAS, 2018–2020) assured additional external quality control all serum analytes.

**PCB COHORT OPFR metabolites**

Urine samples were analyzed for OPFR metabolites at RECETOX (Same as CELSPAC)

Table S2 Ethics committees and funding information of participating HBM4EU aligned studies

| **Study acronym** | **Country** | **Ethics information** |
| --- | --- | --- |
| 3xG | BE | Approved by the ethical committee of University Hospital Antwerp and University Antwerp on 09.11.2010 (Ref N° UA A10-58), amendment for follow-up at 7 years approved on 09.01.2019. Amendment for conducting additional analysis under HBM4EU was approved on 24.08.2020 (Ref N° UA A10-58: 3xG). |
| CELSPAC | CZ | Approved by the Research Ethics Committee of Masaryk University, Czech Republic (Ref. No: EKV-2019-046, 27.05.2019). |
| GerES V | DE | Approved by the Ethics Commission of the Berlin Chamber of Physicians (Eth-14/14) and the Federal Officer for Data Protection and Freedom of Information (III-425/009#0018). |
| OCC | DK | Approved by the Regional Scientific Ethical Review Committee for Southern Denmark (Project ID S-0090130) and the Danish Data Protection Agency (J.No.18/33119). |
| CROME | EL | Approved by the Committee on Ethics and Deontology for Research of AUTH, in the assembly 1/25-10-2018, by request no. Prot. 111256 / 17-09-2018 regarding the research under the project "Cross-Mediterranean Environment and Health Network” and carried out in accordance with the Code of Ethics of Aristotle University of Thessaloniki. |
| ESTEBAN | FR | Approved by the Ile-de-France Protection to person committee on the 06.12.2012 (Internal number: CPP-IDF IX 12-012, EudraCT: 2012-A00459-34). The Committee has examined all provided and requested document (Informed consent, protocol, authorization form from the Ministry of Health, etc). The French Data Protection Agency gave its approval on the 14.02.2013. A Decree of the State Council establishing a processing of personal data relating to biomonitoring, health surveillance and nutrition (The Esteban study) was established after approval of the French Advisory Committee on Information Processing for Research (CCTIRS). The French National Agency for Medicines and Health Products' Safety (ANSM) gave its approval for the use of biological samples and biobanking. |
| NEB II | NO | Approved by The Regional Committees for Medical and Health Research Ethics in Norway, Reference: 2015/1340. |
| SLO CRP | SI | Approved by the National Medical Ethics Committee, Republic of Slovenia (NMEC, number of accordance: 0120-118/2017/3). |
| PCB cohort | SK | Approved by the ethical committee of the Slovak Medical University in Bratislava (No. 01/2018 and 02/2019). |

Table S3 Limits of detection (LOD) and limits of quantification (LOQ) of compounds analyzed in serum/plasma (μg/L).

| Country | PBDE-47 | | PBDE-153 | | PBDE-209 | | αHBCDD | | γHBCDD | | synDP | | antiDP | | TBBPA | | DBDPE | | BEH-TEBP | |
| --- | --- | --- | --- | --- | --- | --- | --- | --- | --- | --- | --- | --- | --- | --- | --- | --- | --- | --- | --- | --- |
|  | LOD | LOQ | LOD | LOQ | LOD | LOQ | LOD | LOQ | LOD | LOQ | LOD | LOQ | LOD | LOQ | LOD | LOQ | LOD | LOQ | LOD | LOQ |
| France | 0.001 | 0.002 | 0.001 | 0.002 | 0.007 | 0.02 | 0.01 | 0.04 | 0.01 | 0.04 | 0.001 | 0.002 | 0.001 | 0.002 | 0.7 | 2 | 0.013 | 0.04 | 0.7 | 2 |
| Greece | 0.0001 | 0.0002 | 0.00003 | 0.0001 | 0.0016 | 0.0052 | 0.0075 | 0.025 | 0.0075 | 0.025 | 0.03 | 0.1 | 0.02 | 0.06 |  |  |  |  |  |  |
| Norway | 0.0003 | 0.0005 | 0.0007 | 0.002 |  |  |  |  |  |  | 0.0002 | 0.0005 | 0.0002 | 0.0005 |  |  |  |  |  |  |
| Slovenia |  |  |  |  |  | 0.05 |  |  |  |  |  | 0.053 |  | 0.04 |  |  |  |  |  |  |

Table S4 Limits of detection (LOD) and limits of quantification (LOQ) of compounds analyzed in urine (μg/L).

| Country |  | BDCIPP | | DPHP | | BCEP | | BCIPP | |
| --- | --- | --- | --- | --- | --- | --- | --- | --- | --- |
|  |  | LOD | LOQ | LOD | LOQ | LOD | LOQ | LOD | LOQ |
| Belgium |  |  | 0.05 |  | 0.1 |  |  |  | 0.4 |
| Czech Republic |  | 0.09 | 0.3 | 0..3 | 0.09 | 0.03 | 1 | 0.09 | 0.3 |
| Germany |  |  | 0.3 |  | 0.15 |  | 0.1 |  | 0.1 |
| Denmark |  |  | 0.05 |  | 0.1 |  |  |  | 0.4 |
| France |  | 0.1 | 0.3 | 0.1 | 0.3 |  |  | 0.2 | 0.7 |
| Norway |  | 0.17 | 0.5 | 0.03 | 0.1 |  |  |  |  |
| Slovenia |  | 0.09 | 0.3 | 0.03 | 0.09 | 0.3 | 1 | 0.09 | 0.3 |
| Slovakia |  | 0.09 | 0.3 | 0.03 | 0.09 | 0.3 | 1 | 0.09 | 0.3 |

Table S5 Detection frequencies of halogenated flame retardants quantified in children’s serum/plasma different countries in this study. Values in green are above 40% detection frequency and were used in statistical analysis. Values in red were excluded from statistical analyses. Compounds that were not studied in a specific country is indicated by n.a.

|  | PBDE-47 | PBDE-153 | PBDE 209 | anti DP | syn DP | αHBCDD | | γHBCDD | | DBDPE | BEH-TEBP | TBBPA |
| --- | --- | --- | --- | --- | --- | --- | --- | --- | --- | --- | --- | --- |
| FR | 45 | 35 | 65 | 32 | 11 | 3 | 0 | | 13 | | 0 | 0 |
| EL | 49 | 78 | 0 | 2 | 0 | 11 | 2 | | 16 | | na | na |
| SI | 15 | 80 | 8 | 41 | 12 | 27 | 5 | | 18 | | na | na |
| NO | 98 | 19 | na | 94 | 100 | na | na | | na | | na | na |

Table S6 Detection frequencies of organophosphate flame retardants quantified in children’s urine different countries in this study. Values in green are above 40% detection frequency and were used in statistical analysis. Values in red were excluded from statistical analyses. Compounds that were not studied in a specific country is indicated by n.a.

|  | BCEP | BCIPP | BDCIPP | DPHP |
| --- | --- | --- | --- | --- |
| FR | na | 31 | 65 | 99 |
| SI | 20 | 18 | 84 | 97 |
| NO | na | na | 17 | 100 |
| DK | na | 7 | 97 | 100 |
| SK | 20 | 29 | 37 | 100 |
| DE | 63 | 53 | 80 | 99 |
| BE | na | 14 | 98 | 99 |
| CZ | 83 | 47 | 57 | 94 |

Table S7 Parameters from single-effect linear regression

| **country** | **compound** | **exposure determinant** | **estimate** | **standard error** | **t value** | **p value (t based)** |
| --- | --- | --- | --- | --- | --- | --- |
| CZ | BCEP | (Intercept) | -0.580 | 0.045 | -12.91 | <0.0001 |
| DE | BCEP | (Intercept) | -0.798 | 0.025 | -31.41 | <0.0001 |
| CZ | BCIPP | (Intercept) | -1.018 | 0.038 | -27.04 | <0.0001 |
| DE | BCIPP | (Intercept) | -0.864 | 0.021 | -41.05 | <0.0001 |
| FR | BCIPP | (Intercept) | 425.310 | 130.366 | 3.26 | 0.001 |
| FR | BCIPP | sampling year | -0.211 | 0.065 | -3.26 | 0.001 |
| FR | BCIPP | home built 1960-1980 | 0.108 | 0.099 | 1.09 | 0.276 |
| FR | BCIPP | home built 1981-2000 | 0.061 | 0.107 | 0.57 | 0.569 |
| FR | BCIPP | home built 2001-2006 | 0.378 | 0.114 | 3.31 | 0.001 |
| FR | BCIPP | home built after 2006 | 0.367 | 0.108 | 3.39 | 0.001 |
| FR | BCIPP | cleaning <1/month | -0.099 | 0.111 | -0.89 | 0.375 |
| FR | BCIPP | cleaning <1/week, >1/month | -0.098 | 0.105 | -0.94 | 0.347 |
| FR | BCIPP | cleaning 2-3 time/week | -0.133 | 0.097 | -1.37 | 0.172 |
| FR | BCIPP | cleaning 7 times/week | -0.498 | 0.150 | -3.31 | 0.001 |
| FR | BCIPP | Drinking tap water | -0.123 | 0.084 | -1.48 | 0.141 |
| FR | BCIPP | Drinking ground water | -0.482 | 0.182 | -2.64 | 0.009 |
| FR | BCIPP | Other drinking water source | -0.245 | 0.122 | -2.02 | 0.045 |
| BE | BDCIPP | (Intercept) | -0.307 | 0.042 | -7.25 | <0.0001 |
| CZ | BDCIPP | (Intercept) | -0.528 | 0.031 | -17.07 | <0.0001 |
| DE | BDCIPP | (Intercept) | -0.174 | 0.021 | -8.44 | <0.0001 |
| DK | BDCIPP | (Intercept) | -0.283 | 0.028 | -10.10 | <0.0001 |
| FR | BDCIPP | (Intercept) | -0.213 | 0.030 | -7.16 | <0.0001 |
| SI | BDCIPP | (Intercept) | -0.193 | 0.024 | -8.12 | <0.0001 |
| SK | BDCIPP | (Intercept) | -183.851 | 77.897 | -2.36 | 0.019 |
| SK | BDCIPP | sampling year | 0.091 | 0.039 | 2.35 | 0.019 |
| EL | BDE-47 | (Intercept) | -3.367 | 0.342 | -9.83 | <0.0001 |
| EL | BDE-47 | male | 0.371 | 0.173 | 2.15 | 0.036 |
| EL | BDE-47 | Vacuuming <1/week, >1/month | -1.066 | 0.321 | -3.32 | 0.002 |
| EL | BDE-47 | Vacuuming 2-3 times/week | -1.022 | 0.375 | -2.73 | 0.009 |
| EL | BDE-47 | Medium income household | -0.296 | 0.184 | -1.61 | 0.114 |
| EL | BDE-47 | High income household | -0.740 | 0.255 | -2.90 | 0.006 |
| FR | BDE-47 | (Intercept) | 425.538 | 127.907 | 3.33 | 0.001 |
| FR | BDE-47 | sampling year | -0.213 | 0.063 | -3.35 | 0.001 |
| FR | BDE-47 | cleaning <1/month | 0.260 | 0.105 | 2.48 | 0.014 |
| FR | BDE-47 | cleaning <1/week, >1/month | 0.162 | 0.103 | 1.57 | 0.118 |
| FR | BDE-47 | cleaning 2-3 time/week | 0.067 | 0.094 | 0.71 | 0.481 |
| FR | BDE-47 | cleaning 7 times/week | 0.125 | 0.133 | 0.94 | 0.349 |
| NO | BDE-47 | (Intercept) | -3.447 | 0.020 | -173.26 | <0.0001 |
| EL | BDE-47 | (Intercept) | -3.364 | 0.318 | -10.58 | <0.0001 |
| EL | BDE-153 | Consumes organic food daily | -0.905 | 0.337 | -2.69 | 0.010 |
| EL | BDE-153 | Consumes canned food <1/month | 0.653 | 0.311 | 2.10 | 0.041 |
| EL | BDE-153 | Consumes canned food <1/week, >1/month | -0.573 | 0.515 | -1.11 | 0.271 |
| EL | BDE-153 | Consumes canned food 4-6 times/week | 0.470 | 0.720 | 0.65 | 0.517 |
| FR | BDE-153 | (Intercept) | -3.499 | 0.025 | -140.36 | <0.0001 |
| SI | BDE-153 | (Intercept) | -3.681 | 0.038 | -95.81 | <0.0001 |
| FR | BDE-209 | (Intercept) | -2.092 | 0.050 | -42.12 | <0.0001 |
| FR | DP | (Intercept) | 209.257 | 69.115 | 3.03 | 0.003 |
| FR | DP | sampling year | -0.106 | 0.034 | -3.08 | 0.002 |
| NO | DP | (Intercept) | -2.488 | 0.031 | -79.33 | <0.0001 |
| SI | DP | (Intercept) | -1.664 | 0.049 | -33.81 | <0.0001 |
| SI | DP | Mother employed | -0.144 | 0.051 | -2.84 | 0.005 |
| BE | DPHP | (Intercept) | 0.391 | 0.026 | 14.82 | <0.0001 |
| CZ | DPHP | (Intercept) | 0.284 | 0.023 | 12.50 | <0.0001 |
| DE | DPHP | (Intercept) | 0.230 | 0.019 | 12.42 | <0.0001 |
| DK | DPHP | (Intercept) | 0.162 | 0.021 | 7.79 | <0.0001 |
| FR | DPHP | (Intercept) | 0.320 | 0.022 | 14.85 | <0.0001 |
| NO | DPHP | (Intercept) | 0.268 | 0.020 | 13.31 | <0.0001 |
| SI | DPHP | (Intercept) | 0.346 | 0.035 | 9.77 | <0.0001 |
| SK | DPHP | (Intercept) | 0.348 | 0.026 | 13.56 | <0.0001 |

Table S8 Flame retardant concentrations in children from previous studies. Concentrations reported in this study are indicated in bold. Studies reporting SG normalized or unadjusted urine concentrations have been converted to median creatinine-normalized units based on the following assumptions: (1) from unadjusted urine to creatinine adjusted, a median ratio of 0.7463 L/g was used (creatinine inverse concentration), determined from the creatinine levels in the CELSPAC study; (2) recalculation from SG normalized concentration to the creatinine according to the median ratio of 0.5814677 L/g (SG standardized creatinine inverse concentration) determined from CELSPAC study. Recalculated values are reported in parentheses below those in the study units.

| Country | Matrix | Age group  (years) | Study start | study end | Unit | Reported as | BCEP | BCIPP | BDCIPP | DPHP | BDE-47 | BDE-153 | BDE-209 | Study |
| --- | --- | --- | --- | --- | --- | --- | --- | --- | --- | --- | --- | --- | --- | --- |
| China | Urine | 0-5 | 2016 | 2016 | ng/ml | GM | 0.67  (0.50) | 0.81  (0.60) | 0.08  (0.06) | 0.25  (0.19) |  |  |  | (Zhang et al., 2018) |
| China | Urine | 8-14 | 2015 | 2015 | µg/L (SG normalised) | Mean | 3.25  (1.04) | 0.21  (0.07) | 0.12 (0.04) | 0.4 (0.13) |  |  |  | (Chen et al., 2018) |
| China | Serum | 9-12 | 2008 | 2008 | ng/g lipid | Median |  |  |  |  | 1.01 | 0.61 | 1.73 | (Zhang et al., 2011) |
| China | Serum | ±10 | 2015 | 2015 | ng/g lipid | Median |  |  |  |  | 4.4 | 2.9 | 95 | (Guo et al., 2016) |
| USA | Urine | 3-6 | 2014 | 2016 | ng/ml (SG corrected) | GM |  | 0.43 (0.25) | 5.63 (3.27) | 2.67 (1.55) |  |  |  | (Phillips et al., 2018) |
| USA | Urine | 3-5 | 2015 | 2015 | ng/ml (SG normalized) | Media |  | 0.9  (0.52) | 2.6  (1.91) | 3.2 (1.86) |  |  |  | (Gibson et al., 2019) |
| USA | Urine | 6-11 | 2013 | 2014 | µg/g (creatine normalized) | GM | 0.855 | 0.344 |  | 2.18 |  |  |  | (Ospina et al., 2018) |
| USA | Serum | <8 | 2008 | 2009 | ng/g lipid | GM |  |  |  |  | 61.8 | 16.8 | 2.76 | (Wu et al., 2015) |
| Canada | Serum | 6-11 | 2007 | 2009 | ng/g lipid | Mean |  |  |  |  | 32 | 14 | 4.3 | (Rawn et al., 2014) |
| Canada | Serum | 1-5 | 2006 | 2010 | ng/g lipid | GM |  |  |  |  | 34.6 | 10.3 |  |  |
| Slovakia | Serum | 6 | 2010 | 2011 | ng/g lipid | Median |  |  |  |  | 0.18 | 0.176 |  | (Drobná et al., 2019) |
| Norway | Urine | 6-11 | 2012 | 2012 | ng/ml (SG corrected) | Mean |  | 1.4 (0.45) | 0.26 (0.08) |  |  |  |  | (Cequier et al., 2015) |
| Norway | Serum | 4-14 | 1998 | 1998 | ng/g lipid | Median |  |  |  |  | 2 | 0.86 |  | (Thomsen et al., 2007) |
| Denmark | Serum | 6-11 | 2011 | 2011 | ng/g lipid | Mean |  |  |  |  | 3.85 | 1.39 |  | (Knudsen et al., 2017) |
| France | Serum | 7-13 | 2014 | 2016 | ng/g lipid | Median |  |  |  |  | **0.36** |  | **8.73** | This study |
| France | Urine | 7-13 | 2014 | 2016 | μg/g creatinine | Median |  |  | **0.58** | **1.92** |  |  |  | This study |
| Greece | Serum | 6-11 | 2020 | 2021 | ng/g lipid | Median |  |  |  |  | **0.03** | **0.09** |  | This study |
| Slovenia | Serum | 7-10 | 2018 | 2018 | ng/g lipid | Median |  |  |  |  |  | **0.29** |  | This study |
| Slovenia | Urine | 7-10 | 2018 | 2018 | μg/g creatinine | Median |  |  | **0.64** | **2.43** |  |  |  | This study |
| Norway | Serum | 8-12 | 2016 | 2017 | ng/g lipid | Median |  |  |  |  | **0.34** |  |  | This study |
| Norway | Urine | 8-12 | 2016 | 2017 | μg/g creatinine | Median |  |  |  | **1.78** |  |  |  | This study |
| Belgium | Urine | 7-8 | 2019 | 2020 | μg/g creatinine | Median |  |  | **0.38** | **2.41** |  |  |  | This study |
| Germany | Urine | 6-12 | 2015 | 2016 | μg/g creatinine | Median | **0.14** | **0.12** | **0.64** | **1.66** |  |  |  | This study |
| Czech Republic | Urine | 11-12 | 2019 | 2019 | μg/g creatinine | Median | **0.22** | **0.10** | **0.26** | **2.06** |  |  |  | This study |
| Slovakia | Urine | 10-13 | 2014 | 2017 | μg/g creatinine | Median |  |  |  | **1.43** |  |  |  | This study |
| Denmark | Urine | 7 | 2018 | 2019 | μg/g creatinine | Median |  |  | **0.49** | **2.21** |  |  |  | This study |

Table S9 Lifestyle factors questionnaire and responses by country

|  |  | **BE** | **CZ** | **DE** | **DK** | **EL** | **FR** | **NO** | **SI** | **SK** |
| --- | --- | --- | --- | --- | --- | --- | --- | --- | --- | --- |
| Sex | Female | 67 | 106 | 150 | 130 | 31 | 194 | 140 | 82 | 167 |
|  | Male | 66 | 89 | 150 | 161 | 24 | 221 | 160 | 67 | 133 |
| Child age (years) | Median age | 7.3 | 11.5 | 9.3 | 7.1 | 10 | 9.4 | 10 | 9.2 | 11.7 |
|  | 6 | 0 | 0 | 15 | 0 | 4 | 2 | 0 | 0 | 0 |
|  | 7 | 81 | 0 | 43 | 290 | 7 | 48 | 12 | 17 | 0 |
|  | 8 | 52 | 0 | 50 | 1 | 5 | 100 | 45 | 36 | 0 |
|  | 9 | 0 | 0 | 49 | 0 | 10 | 61 | 29 | 45 | 0 |
|  | 10 | 0 | 1 | 59 | 0 | 10 | 72 | 112 | 51 | 4 |
|  | 11 | 0 | 103 | 36 | 0 | 19 | 67 | 102 | 0 | 20 |
|  | 12 | 0 | 91 | 48 | 0 | 0 | 54 | 0 | 0 | 253 |
|  | 13 | 0 | 0 | 0 | 0 | 0 | 11 | 0 | 0 | 23 |
| Sampling years | Start | 2019 | 2019 | 2016 | 2019 | 2020 | 2014 | 2016 | 2018 | 2015 |
|  | Finish | 2020 | 2019 | 2016 | 2019 | 2021 | 2016 | 2017 | 2018 | 2017 |
| Years lived in the same house | Median years | 7 | 11 | 7 |  | 9 | 7 | 8 |  |  |
|  | 1 | 21 | 0 | 0 | 0 | 1 | 122 | 71 | 14 | 0 |
|  | 2 | 13 | 0 | 0 | 0 | 4 | 80 | 61 | 45 | 0 |
|  | 3 | 8 | 0 | 0 | 0 | 14 | 68 | 55 | 45 | 0 |
|  | 4 | 7 | 0 | 0 | 0 | 24 | 55 | 12 | 11 | 0 |
|  | 5 | 44 | 0 | 0 | 0 | 12 | 60 | 49 | 31 | 0 |
|  | 6 | 0 | 0 | 0 | 0 | 0 | 0 | 0 | 3 | 0 |
|  | NA | 40 | 195 | 300 | 291 | 0 | 30 | 52 | 0 | 300 |
| Cleaning frequency | Never | 0 | 71 | 0 | 0 | 55 | 91 | 0 | 0 | 0 |
|  | Rarely | 0 | 50 | 0 | 0 | 0 | 69 | 0 | 28 | 0 |
|  | Sometime | 0 | 23 | 0 | 0 | 0 | 81 | 0 | 97 | 0 |
|  | Often | 0 | 15 | 0 | 0 | 0 | 118 | 0 | 20 | 0 |
|  | Very often | 0 | 4 | 0 | 0 | 0 | 0 | 0 | 0 | 0 |
|  | Every day | 0 | 1 | 0 | 0 | 0 | 31 | 0 | 4 | 0 |
|  | NA | 133 | 31 | 300 | 291 | 0 | 25 | 300 | 0 | 300 |
| Vacuum frequency | Rarely | 0 | 0 | 0 | 0 | 4 | 0 | 30 | 12 | 0 |
|  | Sometime | 0 | 0 | 0 | 0 | 43 | 121 | 120 | 50 | 0 |
|  | Often | 0 | 0 | 0 | 0 | 8 | 161 | 98 | 72 | 0 |
|  | Very often | 0 | 0 | 0 | 0 | 0 | 39 | 0 | 0 | 0 |
|  | Every day | 0 | 0 | 0 | 0 | 0 | 56 | 0 | 15 | 0 |
|  | NA | 133 | 195 | 300 | 291 | 0 | 38 | 52 | 0 | 300 |
| Hours indoor | Median hours indoor | NA | 14.4 | NA | NA | 18 | NA | 21 | 23 | NA |
| Local food consumed | Never | 48 | 0 | 0 | 0 | 0 | 0 | 0 | 2 | 0 |
|  | Rarely | 25 | 0 | 0 | 0 | 0 | 0 | 0 | 0 | 0 |
|  | Sometime | 29 | 0 | 0 | 0 | 0 | 0 | 0 | 0 | 0 |
|  | Often | 15 | 0 | 0 | 0 | 0 | 0 | 0 | 9 | 0 |
|  | Very often | 0 | 0 | 0 | 0 | 0 | 0 | 0 | 14 | 0 |
|  | Every day | 16 | 0 | 0 | 0 | 55 | 0 | 0 | 124 | 0 |
|  | NA | 0 | 195 | 300 | 291 | 0 | 415 | 300 | 0 | 300 |
| Organic food consumed | Rarely | 0 | 0 | 0 | 0 | 0 | 137 | 114 | 0 | 0 |
|  | Sometime | 0 | 0 | 0 | 0 | 0 | 67 | 74 | 0 | 0 |
|  | Often | 0 | 0 | 0 | 0 | 0 | 42 | 32 | 0 | 0 |
|  | Very often | 0 | 0 | 0 | 0 | 5 | 33 | 11 | 0 | 0 |
|  | Every day | 0 | 0 | 0 | 0 | 50 | 106 | 11 | 0 | 0 |
|  | NA | 133 | 195 | 300 | 291 | 0 | 30 | 58 | 149 | 300 |
| Seafood consumed | Never | 3 | 23 | 61 | 0 | 23 | 8 | 0 | 97 | 0 |
|  | Rarely | 0 | 25 | 0 | 0 | 22 | 13 | 5 | 19 | 0 |
|  | Sometime | 59 | 43 | 223 | 0 | 9 | 238 | 36 | 33 | 0 |
|  | Often | 70 | 19 | 16 | 0 | 1 | 109 | 173 | 0 | 0 |
|  | Very often | 1 | 5 | 0 | 0 | 0 | 3 | 22 | 0 | 0 |
|  | Every day | 0 | 2 | 0 | 0 | 0 | 0 | 4 | 0 | 0 |
|  | NA | 0 | 78 | 0 | 291 | 0 | 44 | 60 | 0 | 300 |
| Fish consumption | Never | 3 | 17 | 63 | 0 | 0 | 34 | 0 | 35 | 0 |
|  | Rarely | 0 | 31 | 0 | 0 | 8 | 48 | 6 | 20 | 0 |
|  | Sometime | 59 | 51 | 220 | 0 | 26 | 272 | 36 | 79 | 0 |
|  | Often | 70 | 15 | 16 | 0 | 16 | 23 | 177 | 14 | 0 |
|  | Very often | 1 | 3 | 0 | 0 | 5 | 0 | 19 | 1 | 0 |
|  | Every day | 0 | 1 | 0 | 0 | 0 | 0 | 4 | 0 | 0 |
|  | NA | 0 | 77 | 1 | 291 | 0 | 38 | 58 | 0 | 300 |
| Type of drinking water consumed | Bottle water | 0 | 0 | 0 | 0 | 0 | 101 | 0 | 25 | 0 |
|  | Tap water | 0 | 0 | 0 | 0 | 55 | 235 | 291 | 120 | 0 |
|  | Ground water | 0 | 0 | 0 | 0 | 0 | 16 | 0 | 4 | 0 |
|  | Other | 0 | 0 | 0 | 0 | 0 | 38 | 0 | 0 | 0 |
|  | NA | 133 | 195 | 300 | 291 | 0 | 25 | 9 | 0 | 300 |
| Tap water source | Public | 63 | 0 | 292 | 0 | 55 | 0 | 277 | 144 | 0 |
|  | Private well | 0 | 0 | 0 | 0 | 0 | 0 | 23 | 4 | 0 |
|  | Both public and private well | 57 | 0 | 0 | 0 | 0 | 0 | 0 | 1 | 0 |
|  | NA | 13 | 195 | 8 | 291 | 0 | 415 | 0 | 0 | 300 |
| Frequency of fast food | Never | 0 | 5 | 15 | 0 | 9 | 146 | 0 | 9 | 0 |
|  | Rarely | 0 | 36 | 0 | 0 | 16 | 0 | 174 | 29 | 0 |
|  | Sometime | 0 | 106 | 251 | 0 | 22 | 221 | 66 | 110 | 0 |
|  | Often | 0 | 15 | 32 | 0 | 7 | 0 | 0 | 1 | 0 |
|  | Very often | 0 | 0 | 0 | 0 | 1 | 23 | 0 | 0 | 0 |
|  | Every day | 0 | 1 | 2 | 0 | 0 | 0 | 0 | 0 | 0 |
|  | NA | 133 | 32 | 0 | 291 | 0 | 25 | 60 | 0 | 300 |
| Frequency of canned food consumed | Never | 0 | 0 | 0 | 0 | 46 | 33 | 0 | 0 | 0 |
|  | Rarely | 0 | 0 | 0 | 0 | 6 | 0 | 0 | 0 | 0 |
|  | Sometime | 0 | 0 | 0 | 0 | 2 | 300 | 0 | 38 | 0 |
|  | Often | 0 | 0 | 0 | 0 | 0 | 0 | 0 | 32 | 0 |
|  | Very often | 0 | 0 | 0 | 0 | 1 | 57 | 0 | 0 | 0 |
|  | Every day | 0 | 0 | 0 | 0 | 0 | 0 | 0 | 79 | 0 |
|  | NA | 133 | 195 | 300 | 291 | 0 | 25 | 300 | 0 | 300 |
| Vegetarian diet | No | 80 | 118 | 283 | 0 | 55 | 57 | 0 | 149 | 0 |
|  | Yes | 2 | 0 | 13 | 0 | 0 | 1 | 0 | 0 | 0 |
|  | NA | 51 | 77 | 4 | 291 | 0 | 357 | 300 | 0 | 300 |
| Number of cigarettes smoked daily | Median | 0 | 0 |  |  | 0 |  |  | 0 |  |
|  | 0 | 125 | 1 | 272 | 0 | 50 | 0 | 0 | 118 | 0 |
|  | 1 | 0 | 1 | 11 | 0 | 4 | 0 | 0 | 19 | 0 |
|  | 2 | 3 | 0 | 1 | 0 | 1 | 0 | 0 | 3 | 0 |
|  | 3 | 0 | 2 | 0 | 0 | 0 | 0 | 0 | 4 | 0 |
|  | 5 | 2 | 0 | 16 | 0 | 0 | 0 | 0 | 5 | 0 |
|  | NA | 3 | 191 | 0 | 291 | 0 | 415 | 300 | 0 | 300 |
| Age of car (years) | Median | NA | 7 | NA | NA | 10 | NA | 5 | 6 | NA |
| Minutes spent in car daily | Median | 20 | NA | NA | NA | 40 | 107.5 | NA | 20 | NA |
| Birthplace | Europe | 133 | 0 | 298 | 291 | 55 | 397 | 300 | 149 | 300 |
|  | South/Central America | 0 | 0 | 0 | 0 | 0 | 1 | 0 | 0 | 0 |
|  | Africa | 0 | 0 | 0 | 0 | 0 | 12 | 0 | 0 | 0 |
|  | Asia/Middle East | 0 | 0 | 0 | 0 | 0 | 2 | 0 | 0 | 0 |
|  | NA | 0 | 195 | 2 | 0 | 0 | 3 | 0 | 0 | 0 |
| Parity | 1 | 73 | 0 | 0 | 163 | 7 | 0 | 0 | 0 | 118 |
|  | 2 | 48 | 0 | 0 | 100 | 27 | 0 | 0 | 0 | 115 |
|  | 3 | 12 | 0 | 0 | 26 | 18 | 0 | 0 | 0 | 47 |
|  | 4 | 0 | 0 | 0 | 2 | 3 | 0 | 0 | 0 | 16 |
|  | 5 | 0 | 0 | 0 | 0 | 0 | 0 | 0 | 0 | 1 |
|  | NA | 0 | 195 | 300 | 0 | 0 | 415 | 300 | 149 | 3 |
| Degree of urbanization | City | 0 | 0 | 65 | 291 | 46 | 106 | 86 | 0 | 0 |
|  | Town/ suburb | 133 | 0 | 132 | 0 | 8 | 116 | 134 | 0 | 136 |
|  | Rural area | 0 | 0 | 103 | 0 | 1 | 193 | 80 | 149 | 163 |
|  | NA | 0 | 195 | 0 | 0 | 0 | 0 | 0 | 0 | 1 |
| Highest education level of the household | Low education | 1 | 6 | 15 | 40 | 0 | 11 | 0 | 10 | 16 |
|  | Medium education | 19 | 15 | 116 | 151 | 4 | 166 | 14 | 39 | 222 |
|  | High education | 111 | 25 | 169 | 100 | 51 | 237 | 273 | 100 | 45 |
|  | NA | 2 | 149 | 0 | 0 | 0 | 1 | 13 | 0 | 17 |
| Household income level | Low income | 1 | 5 | 0 | 0 | 23 | 19 | 0 | 12 | 0 |
|  | Medium income | 14 | 17 | 0 | 0 | 25 | 162 | 0 | 72 | 0 |
|  | High income | 116 | 16 | 0 | 0 | 7 | 205 | 0 | 48 | 0 |
|  | Do not want to share | 1 | 157 | 0 | 0 | 0 | 26 | 0 | 0 | 0 |
|  | NA | 1 | 0 | 300 | 291 | 0 | 3 | 300 | 17 | 300 |
| Occupation status of mother | employed/ working | 2 | 0 | 58 | 0 | 9 | 0 | 21 | 12 | 0 |
|  | unemployed/ not working | 125 | 0 | 237 | 0 | 46 | 0 | 277 | 135 | 0 |
|  | NA | 6 | 195 | 5 | 291 | 0 | 415 | 2 | 2 | 300 |
| Occupational sector of mother | Armed forces | 0 | 0 | 0 | 0 | 9 | 0 | 0 | 0 | 0 |
|  | Manager | 3 | 0 | 16 | 0 | 2 | 0 | 0 | 4 | 0 |
|  | Professional | 60 | 0 | 55 | 0 | 36 | 0 | 0 | 46 | 0 |
|  | Technician and associated professional | 7 | 0 | 52 | 0 | 0 | 0 | 0 | 17 | 0 |
|  | Clerical support | 35 | 0 | 54 | 0 | 3 | 0 | 0 | 34 | 0 |
|  | Services and sales worker | 13 | 0 | 41 | 0 | 3 | 0 | 0 | 23 | 0 |
|  | skilled agricultural, forestry, and fishery workers | 0 | 0 | 3 | 0 | 0 | 0 | 0 | 3 | 0 |
|  | Crafts and related trade works | 0 | 0 | 3 | 0 | 0 | 0 | 0 | 2 | 0 |
|  | Plant and machine operators and assemblers | 0 | 0 | 3 | 0 | 0 | 0 | 0 | 4 | 0 |
|  | Elementary occupations | 5 | 0 | 10 | 0 | 2 | 0 | 0 | 2 | 0 |
|  | NA | 10 | 195 | 63 | 291 | 0 | 415 | 300 | 14 | 300 |
| Occupation status of father | employed/ working | 0 | 0 | 14 | 0 | 0 | 0 | 8 | 2 | 0 |
|  | unemployed/ not working | 0 | 0 | 260 | 0 | 53 | 0 | 230 | 146 | 0 |
|  | NA | 133 | 195 | 26 | 291 | 2 | 415 | 62 | 1 | 300 |
| Occupational sector of father | Armed forces | 0 | 0 | 0 | 0 | 0 | 0 | 0 | 2 | 0 |
|  | Manager | 0 | 0 | 41 | 0 | 1 | 0 | 0 | 7 | 0 |
|  | Professional | 0 | 0 | 58 | 0 | 36 | 0 | 0 | 25 | 0 |
|  | Technician and associated professional | 0 | 0 | 50 | 0 | 6 | 0 | 0 | 42 | 0 |
|  | Clerical support | 0 | 0 | 15 | 0 | 0 | 0 | 0 | 15 | 0 |
|  | Services and sales worker | 0 | 0 | 15 | 0 | 5 | 0 | 0 | 30 | 0 |
|  | skilled agricultural, forestry, and fishery workers | 0 | 0 | 9 | 0 | 0 | 0 | 0 | 3 | 0 |
|  | Crafts and related trade works | 0 | 0 | 42 | 0 | 2 | 0 | 0 | 9 | 0 |
|  | Plant and machine operators and assemblers | 0 | 0 | 18 | 0 | 3 | 0 | 0 | 13 | 0 |
|  | Elementary occupations | 0 | 0 | 3 | 0 | 0 | 0 | 0 | 0 | 0 |
|  | NA | 133 | 195 | 49 | 291 | 2 | 415 | 300 | 3 | 300 |
| Waste incineration plant within 1 km of the home | No | 0 | 0 | 0 | 0 | 55 | 412 | 0 | 146 | 0 |
|  | Yes | 0 | 0 | 0 | 0 | 0 | 3 | 0 | 3 | 0 |
|  | Don't know/ NA | 133 | 195 | 300 | 291 | 0 | 0 | 300 | 0 | 300 |
| PVC wall present in residence | No | 0 | 0 | 72 | 0 | 55 | 413 | 211 | 145 | 0 |
|  | Yes | 0 | 0 | 0 | 0 | 0 | 2 | 18 | 4 | 0 |
|  | Don't know | 0 | 0 | 0 | 0 | 0 | 0 | 18 | 0 | 0 |
|  | NA | 133 | 195 | 228 | 291 | 0 | 0 | 53 | 0 | 300 |
| PVC floors present in residence | No | 119 | 31 | 68 | 0 | 52 | 337 | 171 | 128 | 181 |
|  | Yes | 14 | 68 | 4 | 0 | 3 | 78 | 57 | 21 | 117 |
|  | Don't know | 0 | 0 | 0 | 0 | 0 | 0 | 19 | 0 | 0 |
|  | NA | 0 | 96 | 228 | 291 | 0 | 0 | 53 | 0 | 2 |
| Carpet floors present in residence | No | 119 | 19 | 20 | 0 | 32 | 394 | 0 | 105 | 247 |
|  | Yes | 14 | 82 | 277 | 0 | 23 | 21 | 0 | 44 | 51 |
|  | Don't know/ NA | 0 | 94 | 3 | 291 | 0 | 0 | 300 | 0 | 2 |
| Renovations to the residence, school, or workplace in the last 2 years | No | 0 | 13 | 0 | 0 | 22 | 180 | 153 | 61 | 212 |
|  | Yes | 0 | 118 | 0 | 0 | 33 | 166 | 96 | 88 | 86 |
|  | Don't know | 0 | 64 | 0 | 0 | 0 | 2 | 0 | 0 | 0 |
|  | NA | 133 | 0 | 300 | 291 | 0 | 67 | 51 | 0 | 2 |
| Smoking | No | 133 | 46 | 300 | 0 | 55 | 0 | 0 | 149 | 292 |
|  | Yes | 0 | 0 | 0 | 0 | 0 | 0 | 0 | 0 | 6 |
|  | NA | 0 | 149 | 0 | 291 | 0 | 415 | 300 | 0 | 2 |
| Exposure to passive smoking | No | 128 | 39 | 272 | 219 | 49 | 365 | 298 | 118 | 154 |
|  | Yes | 5 | 7 | 28 | 72 | 6 | 43 | 2 | 31 | 143 |
|  | NA | 0 | 149 | 0 | 0 | 0 | 7 | 0 | 0 | 3 |
| Alcohol consumption | No | 133 | 0 | 56 | 0 | 55 | 0 | 0 | 0 | 296 |
|  | Yes | 0 | 0 | 4 | 0 | 0 | 0 | 0 | 0 | 2 |
|  | NA | 0 | 195 | 240 | 291 | 0 | 415 | 300 | 149 | 2 |
| How often does the child play with plastic toys? | Never | 0 | 0 | 0 | 0 | 17 | 0 | 94 | 81 | 0 |
|  | Less than once daily | 0 | 0 | 0 | 0 | 11 | 0 | 98 | 40 | 0 |
|  | Daily | 0 | 0 | 0 | 0 | 12 | 0 | 16 | 24 | 0 |
|  | NA | 133 | 195 | 300 | 291 | 15 | 415 | 92 | 4 | 300 |
| Chewing on plastic objects | No | 0 | 0 | 213 | 0 | 39 | 0 | 0 | 0 | 0 |
|  | Yes | 0 | 0 | 87 | 0 | 16 | 0 | 0 | 0 | 0 |
|  | NA | 133 | 195 | 0 | 291 | 0 | 415 | 300 | 149 | 300 |
| Time per day using handheld electronic devices weekday/schoolday | None | 0 | 0 | 0 | 0 | 10 | 0 | 0 | 31 | 0 |
|  | <1 hr/day | 0 | 0 | 0 | 0 | 25 | 0 | 0 | 74 | 0 |
|  | 1-4 hrs/day | 0 | 0 | 0 | 0 | 5 | 0 | 0 | 43 | 0 |
|  | >4 hrs/day | 0 | 0 | 0 | 0 | 15 | 0 | 0 | 1 | 0 |
|  | NA | 133 | 195 | 300 | 291 | 0 | 415 | 300 | 0 | 300 |
| Time per day using handheld electronic devices weekend | None | 0 | 0 | 0 | 0 | 1 | 0 | 0 | 30 | 0 |
|  | <1 hr/day | 0 | 0 | 0 | 0 | 16 | 0 | 0 | 43 | 0 |
|  | 1-4 hrs/day | 0 | 0 | 0 | 0 | 20 | 0 | 0 | 70 | 0 |
|  | >4 hrs/day | 0 | 0 | 0 | 0 | 18 | 0 | 0 | 6 | 0 |
|  | NA | 133 | 195 | 300 | 291 | 0 | 415 | 300 | 0 | 300 |

Never; Rarely: <1 time / month; Sometimes: <= 1 time / week but >= 1 time/month; Often: 2-3 times / week; Very Often: 4-6 times / week; Everyday:> = 7 times / week; NA: Not Answered

Table S10 Lifestyle factors with significant association to flame retardant concentrations. Factors with p-values only were inserted as categorical values, and factors with both p-values and rho-values (ρ) are continuous values

|  | Country | Compound | Confounder | p-value | ρ value | Result |
| --- | --- | --- | --- | --- | --- | --- |
| Socioeconomic factors | | | | | | |
| 1 | DE | BCIPP | Father employment status | 0.006 |  | Higher concentrations where father is employed |
| 3 | NO | BDE-47 | Father employment status | 0.016 |  | Higher concentrations where father is unemployed |
| 4 | DE | DPHP | Household education | 0.005 |  | Lowest in household with low levels of education |
| 5 | DE | DPHP | Father employment status | 0.002 |  | Higher concentrations where father is employed |
| Structural factors | | | | | | |
| 6 | CZ | BCIPP | Renovations | 0.012 |  | Higher concentrations in households that were renovated in the last 2 years |
| 7 | CZ | BDCIPP | PVC floors | 0.013 |  | Higher in households without PVC floors |
| 8 | SI | BDE-153 | PVC wall | 0.036 |  | Higher in houses without PVC walls |
| 9 | FR | BCIPP | Home age | 0.007 |  | Newer homes had higher concentrations |
| Cleaning factors | | | | | | |
| 10 | CZ | BDCIPP | Cleaning | 0.003 |  | Higher concentration in “never” and “rarely” cleaned |
| 11 | FR | BDE-47 | Vacuuming | 0.034 |  | Higher when vacuum frequency is “often” or “every day” |
| 12 | EL | BDE-47 | Vacuuming | 0.009 |  | Higher when vacuum frequency is “never” |
| 13 | SI | BDE-153 | Cleaning | 0.027 |  | Higher concentration in “never” and “rarely” cleaned |
| 14 | CZ | DPHP | Cleaning | <0.001 |  | Higher concentration in “never” and “rarely” cleaned |
| Sex | | | | | | |
| 15 | CZ | BCIPP | Sex | 0.019 |  | Higher concentrations in boys |
| 16 | SI | BDCIPP | Sex | 0.021 |  | Higher concentrations in boys |
| 17 | EL | BDE-47 | Sex | 0.024 |  | Higher concentrations in boys |
| 18 | SI | BDE-153 | Sex | 0.01 |  | Higher concentrations in boys |
| Age | | | | | | |
| 19 | DE | BDCIPP | Age | 0.002 | -0.174 | Concentrations decrease as age increases |
| 20 | FR | BDCIPP | Age | <0.001 | -0.204 | Concentrations decrease as age increases |
| 21 | DE | DPHP | Age | 0.006 | -0.157 | Concentrations decrease as age increases |
| 22 | FR | DPHP | Age | 0.005 | -0.162 | Concentrations decrease as age increases |
| Time in car | | | | | | |
| 23 | BE | BDCIPP | Minutes in car | 0.024 | 0.195 | Concentrations increase as time in car increase |
| 24 | FR | BDCIPP | Minutes in car | 0.001 | 0.189 | Concentrations increase as time in car increase |
| Smoking and food | | | | | | |
| 25 | SI | BDCIPP | Passive smoking | 0.047 |  | Higher where children are exposed to passive smoke at home |
| 26 | BE | DPHP | Fish/seafood | 0.021 |  | Concentrations increase as consumption increases |

**References**

Bastiaensen, M., Xu, F., Been, F., van den Eede, N., Covaci, A., 2018. Simultaneous determination of 14 urinary biomarkers of exposure to organophosphate flame retardants and plasticizers by LC-MS/MS. Anal Bioanal Chem 410, 7871–7880. https://doi.org/10.1007/s00216-018-1402-2

Caspersen, I.H., Kvalem, H.E., Haugen, M., Brantsæter, A.L., Meltzer, H.M., Alexander, J., Thomsen, C., Frøshaug, M., Bremnes, N.M.B., Broadwell, S.L., Granum, B., Kogevinas, M., Knutsen, H.K., 2016. Determinants of plasma PCB, brominated flame retardants, and organochlorine pesticides in pregnant women and 3 year old children in The Norwegian Mother and Child Cohort Study. Environ Res 146, 136–144. https://doi.org/10.1016/j.envres.2015.12.020

Cequier, E., Marcé, R.M., Becher, G., Thomsen, C., 2014. A high-throughput method for determination of metabolites of organophosphate flame retardants in urine by ultra performance liquid chromatography-high resolution mass spectrometry. Anal Chim Acta 845, 98–104. https://doi.org/10.1016/j.aca.2014.06.026

Cequier, E., Sakhi, A.K., Haug, L.S., Thomsen, C., 2016. Development of an ion-pair liquid chromatography–high resolution mass spectrometry method for determination of organophosphate pesticide metabolites in large-scale biomonitoring studies. J Chromatogr A 1454, 32–41. https://doi.org/10.1016/j.chroma.2016.05.067

Cequier, E., Sakhi, A.K., Marcé, R.M., Becher, G., Thomsen, C., 2015. Human exposure pathways to organophosphate triesters - A biomonitoring study of mother-child pairs. Environ Int 75, 159–165. https://doi.org/10.1016/j.envint.2014.11.009

Chen, Y., Fang, J., Ren, L., Fan, R., Zhang, J., Liu, G., Zhou, L., Chen, D., Yu, Y., Lu, S., 2018. Urinary metabolites of organophosphate esters in children in South China: Concentrations, profiles and estimated daily intake. Environmental Pollution 235, 358–364. https://doi.org/10.1016/j.envpol.2017.12.092

Drobná, B., Fabišiková, A., Čonka, K., Gago, F., Oravcová, P., Wimmerová, S., Oktapodas Feiler, M., Šovčíková, E., 2019. PBDE serum concentration and preschool maturity of children from Slovakia. Chemosphere 233, 387–395. https://doi.org/10.1016/j.chemosphere.2019.05.284

European Commission, 2021a. GerES V - German Environmental Survey 2014-2017 [WWW Document]. https://doi.org/10.1007/S10654-019-00500-X

European Commission, 2021b. ESTEBAN - Environment, Health, Biomonitoring, physical Activity, Nutrition : Esteban [WWW Document]. URL https://ipchem.jrc.ec.europa.eu/#showmetadata/ESTEBAN (accessed 6.8.22).

Frederiksen, M., Andersen, H.V., Haug, L.S., Thomsen, C., Broadwell, S.L., Egsmose, E.L., Kolarik, B., Gunnarsen, L., Knudsen, L.E., 2020. PCB in serum and hand wipes from exposed residents living in contaminated high-rise apartment buildings and a reference group. Int J Hyg Environ Health 224. https://doi.org/10.1016/j.ijheh.2019.113430

Gibson, E.A., Stapleton, H.M., Calero, L., Holmes, D., Burke, K., Martinez, R., Cortes, B., Nematollahi, A., Evans, D., Anderson, K.A., Herbstman, J.B., 2019. Differential exposure to organophosphate flame retardants in mother-child pairs. Chemosphere 219, 567–573. https://doi.org/10.1016/j.chemosphere.2018.12.008

Guo, W., Holden, A., Smith, S.C., Gephart, R., Petreas, M., Park, J.S., 2016. PBDE levels in breast milk are decreasing in California. Chemosphere 150, 505–513. https://doi.org/10.1016/j.chemosphere.2015.11.032

Hertz-Picciotto, I., Trnovec, T., Kočan, A., Charles, M.J., Čižnar, P., Langer, P., Sovčikova, E., James, R., 2003. PCBs and early childhood development in Slovakia: Study design and background. Fresenius Environ Bull 12, 208–214.

Knudsen, L.E., Hansen, P.W., Mizrak, S., Hansen, H.K., Mørck, T.A., Nielsen, F., Siersma, V., Mathiesen, L., 2017. Biomonitoring of Danish school children and mothers including biomarkers of PBDE and glyphosate. Rev Environ Health 32, 279–290. https://doi.org/10.1515/reveh-2016-0067

Ospina, M., Jayatilaka, N.K., Wong, L.Y., Restrepo, P., Calafat, A.M., 2018. Exposure to organophosphate flame retardant chemicals in the U.S. general population: Data from the 2013–2014 National Health and Nutrition Examination Survey. Environ Int 110, 32–41. https://doi.org/10.1016/j.envint.2017.10.001

Palát, J., Kukučka, P., Codling, G.P., Price, E.J., Janků, P., Klánová, J., 2022. Application of 96-well plate SPE method for analysis of persistent organic pollutants in low volume blood serum samples. Chemosphere 287, 132300. https://doi.org/10.1016/j.chemosphere.2021.132300

Phillips, A.L., Hammel, S.C., Hoffman, K., Lorenzo, A.M., Chen, A., Webster, T.F., Stapleton, H.M., 2018. Children’s residential exposure to organophosphate ester flame retardants and plasticizers: Investigating exposure pathways in the TESIE study. Environ Int 116, 176–185. https://doi.org/10.1016/j.envint.2018.04.013

Rawn, D.F.K., Ryan, J.J., Sadler, A.R., Sun, W.F., Weber, D., Laffey, P., Haines, D., Macey, K., van Oostdam, J., 2014. Brominated flame retardant concentrations in sera from the Canadian Health Measures Survey (CHMS) from 2007 to 2009. Environ Int 63, 26–34. https://doi.org/10.1016/j.envint.2013.10.012

Stajnko, A., Snoj Tratnik, J., Kosjek, T., Mazej, D., Jagodic, M., Eržen, I., Horvat, M., 2020. Seasonal glyphosate and AMPA levels in urine of children and adolescents living in rural regions of Northeastern Slovenia. Environ Int 143. https://doi.org/10.1016/j.envint.2020.105985

Svarcova, A., Lankova, D., Gramblicka, T., Stupak, M., Hajslova, J., Pulkrabova, J., 2019. Integration of five groups of POPs into one multi-analyte method for human blood serum analysis: An innovative approach within biomonitoring studies. Science of the Total Environment 667, 701–709. https://doi.org/10.1016/j.scitotenv.2019.02.336

Thomsen, C., Liane, V.H., Becher, G., 2007. Automated solid-phase extraction for the determination of polybrominated diphenyl ethers and polychlorinated biphenyls in serum-application on archived Norwegian samples from 1977 to 2003. J Chromatogr B Analyt Technol Biomed Life Sci 846, 252–263. https://doi.org/10.1016/j.jchromb.2006.09.011

Wu, X., Bennett, D.H., Moran, R.E., Sjödin, A., Jones, R.S., Tancredi, D.J., Tulve, N.S., Clifton, M.S., Colón, M., Weathers, W., Hertz-Picciotto, I., 2015. Polybrominated diphenyl ether serum concentrations in a Californian population of children, their parents, and older adults: An exposure assessment study. Environ Health 14. https://doi.org/10.1186/s12940-015-0002-2

Zhang, B., Lu, S., Huang, M., Zhou, M., Zhou, Z., Zheng, H., Jiang, Y., Bai, X., Zhang, T., 2018. Urinary metabolites of organophosphate flame retardants in 0–5-year-old children: Potential exposure risk for inpatients and home-stay infants. Environmental Pollution 243, 318–325. https://doi.org/10.1016/j.envpol.2018.08.051

Zhang, X., Ruan, X., Yan, M., Zhao, Y., Wei, W., Qin, Z., Yang, Y., Xu, H., Li, Y., 2011. Polybrominated diphenyl ether (PBDE) in blood from children (age 9-12) in Taizhou, China. Journal of Environmental Sciences 23, 1199–1204. https://doi.org/10.1016/S1001-0742(10)60553-0
